# Manual therapy for the pediatric population: a systematic review

**DOI:** 10.1186/s12906-019-2447-2

**Published:** 2019-03-13

**Authors:** Carol Parnell Prevost, Brian Gleberzon, Beth Carleo, Kristian Anderson, Morgan Cark, Katherine A. Pohlman

**Affiliations:** 1 Palmer College of Chiropractic, 4777 City Center Parkway, Port Orange, FL 32129 USA; 2 Canadian Memorial Chiropractic College, 6100 Leslie St., North York, ON M2H 3J1 Canada; 3 Performance Chiropractic, 4350 South Washington Street Suite 100, Grand Forks, ND 58201 USA; 4 Parker University, 2540 Walnut Hill Lane, Dallas, TX 75229 USA

**Keywords:** Pediatric, Manual therapy, Chiropractic, Osteopathic, Systematic review

## Abstract

**Background:**

This systematic review evaluates the use of manual therapy for clinical conditions in the pediatric population, assesses the methodological quality of the studies found, and synthesizes findings based on health condition. We also assessed the reporting of adverse events within the included studies and compared our conclusions to those of the UK Update report.

**Methods:**

Six databases were searched using the following inclusion criteria: children under the age of 18 years old; treatment using manual therapy; any type of healthcare profession; published between 2001 and March 31, 2018; and English. Case reports were excluded from our study. Reference tracking was performed on six published relevant systematic reviews to find any missed article. Each study that met the inclusion criteria was screened by two authors to: (i) determine its suitability for inclusion, (ii) extract data, and (iii) assess quality of study.

**Results:**

Of the 3563 articles identified, 165 full articles were screened, and 50 studies met the inclusion criteria. Twenty-six articles were included in prior reviews with 24 new studies identified. Eighteen studies were judged to be of high quality. Conditions evaluated were: attention deficit hyperactivity disorder (ADHD), autism, asthma, cerebral palsy, clubfoot, constipation, cranial asymmetry, cuboid syndrome, headache, infantile colic, low back pain, obstructive apnea, otitis media, pediatric dysfunctional voiding, pediatric nocturnal enuresis, postural asymmetry, preterm infants, pulled elbow, suboptimal infant breastfeeding, scoliosis, suboptimal infant breastfeeding, temporomandibular dysfunction, torticollis, and upper cervical dysfunction. Musculoskeletal conditions, including low back pain and headache, were evaluated in seven studies. Twenty studies reported adverse events, which were transient and mild to moderate in severity.

**Conclusions:**

Fifty studies investigated the clinical effects of manual therapies for a wide variety of pediatric conditions. Moderate-positive overall assessment was found for 3 conditions: low back pain, pulled elbow, and premature infants. Inconclusive unfavorable outcomes were found for 2 conditions: scoliosis (OMT) and torticollis (MT). All other condition’s overall assessments were either inconclusive favorable or unclear. Adverse events were uncommonly reported. More robust clinical trials in this area of healthcare are needed.

**Trial registration:**

PROSPERA registration number: CRD42018091835

## Background

Parents consult complementary and alternative medicine (CAM) providers for a wide variety of pediatric conditions [1, 2]. In addition to botanical medicines and supplements, some seek manual therapy including soft tissue therapy, mobilization and high velocity low amplitude manipulations directed to the spine and peripheral joints. The United States (US) Department of Health and Human Services conducts a population-based survey and creates the National Health Interview Statistics (NHIS) reports on the use of CAM with children ages 4–17 every 5 years with results published in 2007 and 2012. Overall, approximately 12% of children used a CAM modality the previous year [1, 2].

Manual therapy is a CAM therapy regulated for use among many professions (e.g., doctor of osteopathy, medical doctors and physical therapists), but doctors of chiropractic (DCs) are the most likely profession to use manual therapy on a regular basis [3]. According to a recent job analysis of the overall DC profession, 17.1% of chiropractic patients are 17 years of age or less; this increases to 38.7% among DCs who have specialized in pediatrics [3, 4]. Ndetan et al. conducted a sub-analysis of the 2007 NHIS data for chiropractic and/or osteopathic manipulation use and found that 3.3% of US children had received chiropractic or osteopathic manipulation the previous year [5]. Most commonly, children were between 12 and 18 years of age and received care for back or neck pain.

Concerns regarding manual therapy, specifically manipulation [6], have led to complications identified in the literature. However, no prospective population-based active surveillance have been conducted [7]. Serious events are rare, but may be related to high-velocity extension and rotational spinal manipulation [8]. The serious events identified in mostly retrospective studies commonly occurred with patients that had preexisting underlying pathology, which emphasizes the need for a thorough history and physical examination so that abnormal findings are identified prior to manual therapy in a child [7–9].

Six systematic reviews have previously been conducted to evaluate the use of manual therapy for pediatric health conditions [9–14]. These reviews ranged in manual therapy definitions from high-velocity variable amplitude to profession-specific manipulative therapy. Nonetheless, all reviews concluded that this is a paucity of evidence for the effectiveness of manual therapy for conditions within the pediatric population, especially for musculoskeletal conditions. The purpose of this systematic review was to evaluate the use of manual therapy for clinical conditions in the pediatric population, assess the methodological quality of the studies found, and synthesize findings based on health condition. We also assessed the reporting and incidence of adverse events within the included studies. Additionally, we compared conclusions to Clar et al.’s UK Update manuscript [10].

## Methods

This study was registered at the PROSPERA - Center for Reviews and Dissemination, University of York, York, U.K. on March 28, 2018. Details of the protocol for this systematic review were registered on PROSPERO and can be accessed at https://www.crd.york.ac.uk/prospero/display_record.php?RecordID=91835.

### Search strategy

A comprehensive search of the literature was performed by three independent librarians at three different educational institutions. The databases stated in Table 1 were searched for English manuscripts published between 2001 through March 31, 2018. Data mining and reference tracking of the six previously published systematic reviews were performed for relevant papers. No condition terms were included to keep the search as broad as possible. The list of search terms and keywords used in the search are included in Table 1.

**Table 1:** Databases searched: PubMed, Cochrane Library, Medline complete, CINAHL complete, ScienceDirect, McCoy Press, Index to Chiropractic Literature, and National Guideline Clearinghouse

Chiropractic	AND	pediatric*
Chiropractic	AND	child*
Chiropractic	AND	adolescent*
Manipulation, chiropractic (MeSH heading)	AND	(pediatric*, child*, adolescent*)
Manipulation, orthopedic (MeSH heading)	AND	(pediatric*, child*, adolescent*)
Manipulation, osteopathic (MeSH heading)	AND	(pediatric*, child*, adolescent*)
Osteopath*	AND	(pediatric*, child*, adolescent*)
Orthopedic manipulation	AND	(pediatric*, child*, adolescent*)
Orthoped*	AND	(pediatric*, child*, adolescent*)
Pediatric manual therapy	AND	(pediatric*, child*, adolescent*)
Ped MT	AND	(pediatric*, child*, adolescent*)
Spinal manipulative therapy	AND	(pediatric*, child*, adolescent*)
SMT	AND	(pediatric*, child*, adolescent*)

### Eligibility criteria

Studies were eligible for inclusion if they were full text reports of RCTs (no abstracts). Feasibility studies without outcome measures were not included in this review. For observational studies, the Agency for Healthcare Research and Quality’s (AHRQ) *Assessing Risk of Bias and Confounding in Observational Studies of Interventions or Exposures* was utilized to determine study type with non-comparative (case report or case series without pre and post measurements) and cross-sectional studies excluded [15]. Additional eligibility criteria were that a study had to include children under the age of 18 who were treated with manual therapy (definitions and abbreviations shown in Table 2) of any type from any health care professional for any condition.

**Table 2:** Abbreviations and definitions used for this study

SMT (Spinal Manipulative Therapy)	A procedure involving an high velocity, low amplitude (HVLA) thrust beyond the passive range of motion into the para-physiological space, but within the limits of anatomic integrity [71]^p10^, [72]^p142–143^, [73]. It is a bimanual motor skill involving various levels of interlimb coordination and postural control combined with a timely weight transfer and is characterized by a HVLA thrust that typically results in joint cavitation [74]. SMT is highly adaptive and context-dependent, meaning the amount of force delivered to the patient must take into account clinically relevant pathologies as well as anthropomorphic differences between the doctor and patient [73].The safe delivery of SMT requires consideration with respect to preload, speed of force production, peak amplitude of force delivered, duration of impulse/thrust delivered, doctor position, patient positioning, and line of drive (direction of thrust) [71, 74].
Mobilization	A low velocity, low amplitude (LVLA) oscillation procedure, within the active or passive ranges of motion [71]^p18^, [72]^p142^.
OMT (Osteopathic Manipulative Therapy)	Involves physical manipulation of various tissues and parts of the body that includes soft tissue massage and stretch, strain-counter-strain, articulation, high velocity thrust, gentle low amplitude mobilizations and neuromuscular techniques [49]^p1–2^. In some instances OMT is better classified as a mobilization [71]^p18^ .
CST (Cranial-Sacral Therapy)	A group of manual procedures directed to the sutures of the skull designed to enhance the functioning of the membranes, tissues, fluids, and bones surrounding or associated with the brain and spinal cord. It is postulated that low-force pressure can influence the vitality of the Cranial Rhythmic Impulse created by the flow of cerebrospinal fluid as it moves from the ventricles of the skull to the sacrum within the spinal cord [71]^p123–136^.
CMT (Chiropractic Manipulative Therapy)	Synonymous with SMT, but performed by a doctor of chiropractic.
VOMT (Visceral Osteopathic Manipulation)	A manual therapy directed to various organs of the body to aid in smooth muscle function, influence somatic biomechanics and body fluid mechanics [49]^p251–252^.
Instrument-assisted manipulation	The use of any number of different types of hand held instruments used to provide a manipulation-type force.
MT (Manual Therapy)	Any of the above.

### Study selection, data extraction, & summary assessment

Two independent reviewers evaluated the studies identified by the searches for potential inclusion in our study. They applied the inclusion/exclusion criteria to the studies identified by first screening the abstracts and then the full text of any studies appearing to fulfill the inclusion criteria. Any discrepancies as to whether or not to include a study was resolved by a third independent evaluator. Data extraction was conducted by an independent reviewer using an *a priori* designed data extraction form with a second reviewer validating the findings.

An overall result summary assessment was determined for each study based on their results as either: “improvement” (manual therapy appeared to be effective in the intervention group), “no improvement” (manual therapy did not appear to be effective in the intervention group), or “no difference” (results appeared to be the same in the intervention group as compared to a different intervention, sham intervention or control group).

### Quality assessment-individual studies

The quality assessment process was conducted by an independent reviewer and validated by a second randomly assigned reviewer. Disagreements for each criterion were settled through discussion with a third reviewer. Two different assessment tools were utilized to assess the quality of the RCTs and observational studies included in this review. The Cochrane Risk of Bias tool, consisting of 7 domains, was used to assess the risk of bias of the RCTs [16]. The domains were:

- adequate sequence generation,
- allocation concealment,
- patient blinding,
- assessor blinding,
- addressing of incomplete data,
- selective outcome reporting, and
- other sources of bias.

The tool used to assess observational studies was the same used to evaluate the observational study design [15]. This AHRQ tool consists of 9 domains:

- inclusion/exclusion criteria variances across groups (cohort studies only),
- recruitment strategies for groups (cohort studies only),
- appropriate, selection of comparison groups (cohort studies only),
- blinding outcome assessor to intervention,
- use of valid and reliable outcome tools,
- length of follow-up variances across study groups,
- missing important primary outcomes,
- missing harms or adverse events, and
- account of any confounding variables.

We omitted the following questions from the AHRQ assessment for the following reasons. Questions 4 (Does the study fail to account for important variations in the execution of the study from the proposed protocol?) and 12 (Any attempt to balance the allocation between the groups or match groups (e.g., through stratification, matching, propensity scores)?) as these were not relevant for our body of literature. Question 8 (In cases of high loss to follow-up (or differential loss to follow-up), was the impact assessed (e.g., through sensitivity analysis or other adjustment method)?) as our included studies did not have this level of statistical analysis involved. And question 11 (Are results believable taking study limitations into consideration?) as we felt this question was too subjective [15, 17].

The study’s overall quality score was then determined to be: low quality study if the score was between 0 and 33.3%, medium quality, if the score was between 33.4 and 66.6%, and high quality if the score was above 66.6%.

### Quality assessment-overall conditions

We employed the same criteria to summarize the overall strength of evidence for the studies by conditions to be consistent with the UK Update/Clar et al. report [10], which used an adapted version from the US Preventive Services Task Force. This report, along with Clar et al. reports, summarized the overall strength/quality of evidence as: “high-quality positive/negative”, “medium-quality positive/negative”, or “inconclusive evidence favorable/non-favorable/unclear” [10]. The overall evidence grading system used allows the evidence to be grouped into three categories based upon its strength: high quality evidence, moderate quality evidence, or inconclusive evidence. The definitions of these three categories are listed below:

#### High quality evidence

The evidence comes from at least 2 RCTs and is considered high quality due to low risk of bias. As a result, the conclusion is unlikely to be affected by future studies.

#### Moderate quality evidence

The evidence comes from at least 1 high-quality RCT (with sufficient statistical validity) OR at least 2 higher-quality RCTs (with some inconsistency) OR at least 2 consistent lower-quality RCTs.

#### Low quality (inconclusive) evidence

The available evidence is insufficient to determine effectiveness. If all papers showed improvement they are classified as “Favorable”. If all papers failed to show improvement they are classified as “Unfavorable”. If all papers showed a mix of improvement, lack of improvement, or no difference they are classified as “Unclear”. Note that observational studies cannot be rated higher than “Inconclusive (unclear)”, as observational studies are not designed to show effectiveness.

## Results

### Search results

**Fig. 1:**
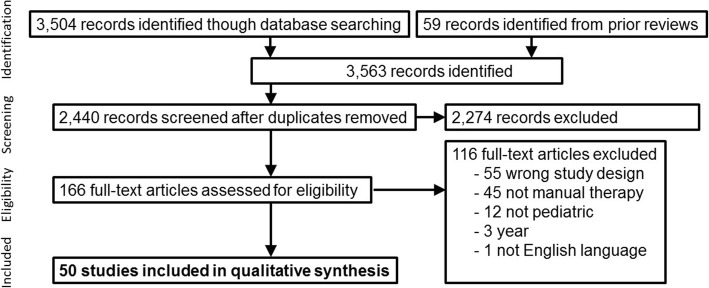
Study selection flow diagram (PRISMA style)

As shown in Fig. 1, the initial database searches generated a total of 3563 records (2440 after deduplication). Of which, 166 full articles were assessed in detail. One hundred sixteen of the articles were excluded. Of the 50 included articles, 32 were RCTs and 18 were observational studies. Table 3 provides a summary of the studies along with the details, sample sizes, quality, results of the study and an overall summary. This table also compares the overall summary from Clar et al.’s UK Update study [10]. These studies are then summarized by study design (RCT and observational) in Table 4 and 5, respectively, with the individual quality assessment criteria outlined.

**Table 3:** Overall summary in comparison with the UK Update report

Clinical condition	UK update (1) summary	Studies in current review	Intervention	Citations	Quality	Sample size	Results of study	Current study overall summary
Gastrointestinal Conditions								
Constipation	Not evaluated	1 OBS	OMT	Tarsuslu, 2009 [18]	Medium	13	No difference	Inconclusive (unclear)
Infantile Colic	Inconclusive/ favorable	3 RCT 1 OBS	CMT	Miller, 2012 [19] Wiberg, 2010 [20] Browning, 2008 [21] Olafsdottir, 2001 [22]	High Low High High	104 749 43 86	Improvement No improvement No difference No difference	Inconclusive (unclear)
Infantile Colic	Inconclusive/ favorable	1 RCT	OMT/CST	Hayden, 2006 [23]	Medium	28	Improvement	Inconclusive (favorable)
Pediatric dysfunctional voiding	Inconclusive/ favorable	1 RCT	OMT	Nemett, 2008 [24]	Medium	21	Improvement	Inconclusive (favorable)
Pediatric nocturnal enuresis	Inconclusive/ favorable	1 OBS	CMT	van Poecke, 2009 [25]	Medium	33	Improvement	Inconclusive (unclear)
Suboptimal infant breastfeeding	Not evaluated	2 OBS	CMT/CST	Miller, 2009 [26] Vallone, 2004 [27]	Medium Low	114 25	Improvement Improvement	Inconclusive (favorable)
Musculoskeletal Conditions								
Clubfoot	Not evaluated	1 RCT	MT	Nilgun, 2011 [28]	Low	29	Improvement	Inconclusive (favorable)
Cuboid Syndrome	Not evaluated	1 OBS	MT	Jennings, 2005 [29]	Medium	2	Improvement	Inconclusive (unclear)
Headache	Not evaluated for pediatrics	1 OBS	OMT	Przekop, 2016 [30]	Medium	83	Improvement	Inconclusive (unclear)
Headache	Not evaluated for pediatrics	1 RCT	MT	Borusiak, 2010 [31]	Medium	52	No difference	Inconclusive (unclear)
Headache	Not evaluated for pediatrics	1 OBS	CMT	Marchand, 2009 [32]	Low	13	Improvement	Inconclusive (unclear)
Low Back Pain	Not evaluated	1 RCT 1 OBS	CMT	Evans, 2018 [33] Hayden J, 2003 [36]	High Medium	185 54	Improvement Improvement	Moderate (favorable)
Low back pain	Not evaluated	1 OBS 1 RCT	MT	Walston, 2016 [34] Selhorst, 2015 [35]	Medium Medium	3 35	Improvement No difference	Inconclusive (unclear)
Pulled (Nursemaid’s) Elbow	Not evaluated	2 RCT	MT	Garcia-Mata, 2014 [37] Bek, 2009 [38]	Medium Medium	115 66	Improvement Improvement	Moderate (favorable)
Temporomandibular Joint Dysfunction	Not evaluated for pediatrics	1 RCT	OMT	Monaco, 2008 [39]	Low	28	Improvement	Inconclusive (favorable)
Respiratory Conditions								
Asthma	Not evaluated for pediatrics	1 RCT	OMT	Guiney, 2005 [40]	Medium	140	Improvement	Inconclusive (favorable)
Asthma	Not evaluated for pediatrics	1 RCT	CMT	Bronfort, 2001 [41]	High	34	No improvement	Inconclusive (unclear)
Obstructive apnea	Not evaluated	1 RCT	OMT	Vandenplas, 2008 [42]	Medium	34	Improvement	Inconclusive (favorable)
Otitis Media	Inconclusive/ unclear	3 RCT 1 OBS	OMT	Steele, 2014 [43] Wahl, 2008 [44] Degenhardt, 2006 [45] Mills, 2003 [47]	Medium High Medium High	34 90 8 57	Improvement No difference Improvement Improvement	Inconclusive (favorable)
Otitis Media	Not evaluated	1 OBS	CMT	Zhang, 2004 [46]	Medium	22	Improvement	Inconclusive (unclear)
Special Needs								
ADHD	Inconclusive/ unclear	1 RCT	OMT	Accorsi, 2014 [48]	High	28	Improvement	Inconclusive (favorable)
Autism	Not evaluated	1 OBS	VOMT	Bramati-Castellarian, 2016 [49]	Medium	49	Improvement	Inconclusive (unclear)
	Not evaluated	1 RCT	CMT	Khorshid, 2006 [50]	Low	14	Improvement	Inconclusive (favorable)
Cerebral Palsy	Inconclusive/ unclear	3 RCT	OMT	Wyatt, 2011 [51] Duncan, 2008 [53] Duncan, 2004 [52]	High High Low	142 55 50	No improvement Improvement Improvement	Inconclusive (unclear)
Preterm infants	Inconclusive/ unclear	4 RCT	OMT/CST	Raith, 2015 [54] Cerretelli, 2015 [55] Pizzolorusso, 2014 [56] Cerretelli, 2013 [57]	High High High High	30 695 110 110	No difference Improvement Improvement Improvement	Inconclusive/unclear for general movement Moderate (favorable) length of stay and hospital costs
Structural Conditions								
Cranial asymmetry	Not evaluated	1 RCT	MT/CST	Cabrera-Martos, 2016 [58]	High	46	Improvement	Inconclusive (favorable)
	Not evaluated	1 OBS	OMT	Lessard, 2011 [59]	Medium	12	Improvement	Inconclusive (unclear)
Postural Asymmetry	Not evaluated	1 RCT	OMT/CST	Phillippi, 2006 [60]	High	32	Improvement	Inconclusive (favorable)
Scoliosis	Not evaluated	1 RCT 3 OBS	CMT	Byun, 2016 [61] Rowe, 2006 [62] Morningstar, 2004 [63] Lantz, 2001 [64]	Medium High Low Medium	5 6 6 42	Improvement No difference Improvement No improvement	Inconclusive (unclear)
Scoliosis	Not evaluated	1 RCT	OMT	Hasler, 2010 [65]	High	20	No improvement	Inconclusive (unfavorable)
Torticollis	Not evaluated	1 RCT	MT	Haugen, 2011 [66]	Medium	32	No difference	Inconclusive (unfavorable)
Upper cervical dysfunction	Not evaluated	1 OBS	MT	Saedt, 2017 [67]	High	307	Improvement	Inconclusive (unclear)

**Table 4:** Quality rating of randomized controlled trials

Author/year	Condition sample size (n)	Results summary	Intervention	Selection bias: random	Selection bias: allocation	Performance bias: blinding of personnel and participants	Detection bias: blinding of outcome assessment	Attrition bias: incomplete outcome data	Reporting bias: selective reporting	Other bias: anything else, ideally pre-specified	Overall quality rating
Gastrointestinal/Urinary											
Miller J, et al. 2012 [19]	Infantile Colic (*n* = 104)	Improvement	CMT	L computer generated permutated blocks	L sealed in sequentially numbered opaque envelopes	L envelopes revealed to treating provider before treatment, 1 of 3 groups parents knew infants were being treated	U-PY two of three groups of parents blinded to treatment, data extractor blinded to teratment	H per protocol analysis conducted	L all outcomes reported	U-PN “parent diagnosis”, selective nature of diary	High
Browning M & Miller J, 2008 [21]	Infantile Colic (*n* = 43)	No difference	CMT	L computer generated	H not stated	L blinding of both parents and patients	L independent observer binded to treatment	L all outcomes reported	L all outcomes reported	H strict inclusion criteria, small study size, inexperienced iterns	High
Hayden C & Mullinger B, 2006 [23]	Infantile Colic (*n* = 28)	Improvement	OMT/CST	L random number table	U-PY random table number utilized but not discussed	H patients and providers not blinded	H outcome assessors unblinded	H 2 withdrew and not included in analysis	L all outcomes reported	U-PN small study size, lack of standardized treatment	Medium
Olafsdottir E, et al. 2001 [22]	Infantile Colic (*n* = 86)	No difference	CMT	H “randomized” not described	U-PY “sealed” envelopes	L parents and providers blinded	L outcome assessor blinded	L intention to treat analysis	L all outcomes reported	U-PY small sample size	High
Musculoskeletal											
Nemett D, et al. 2008 [24]	Pediatric Dysfunctional Voiding (*n* = 21)	Improvement	OMT	U-PY stated “randomized assigned” with no further description	H nothing stated	H nothing stated	H only primary outcome assessor blinded	H per protocol analysis conducted	L all expected outcomes reported, secondary outcome not initially evaluated in control group per protocol	L study appears free of other sources of bias	Medium
Nilgun B, et al. 2011 [28]	Idiopathic Clubfoot (*n* = 29)	Improvement	MT	H randomized by travel and physical abilities	H not concealed	H parents, patients, therapists not blinded	H outcome assessor not blinded	L all outcomes reported	L all outcomes reported	H pilot study only	Low
Borusiak P, et al. 2010 [31]	Cervicogenic HA (*n* = 52)	No difference	MT	L computer generated	L sequentially numbered identical opaque envelopes	L parents, patients and pediatrician blinded	U-PY pre-established analysis plan not described	H per protocol analysis conducted	L all outcomes reported	H small sample size, clinical effect of sham, observational bias	Medium
Evans R, et al. 2018 [33]	Subacute and Chronic LBP (*n* = 185)	Improvement	CMT	L computerized dynamic allocation (rank-order minization) system	L sealed in sequentially numbered opaque envelopes	H patients and providers not blinded	L outcome assessor blinded	L all outcomes reported	L all outcomes reported	L study appears free of other sources of bias	High
Selhorst M & Selhorst B, 2015 [35]	Mechanical LBP (*n* = 35)	No difference	MT	H not described	H not described	U-PY blinding of patients, exercise therapist, no blinding of manual therapist	L all outcomes patient self-report blinded	H per protocol anaylsis conducted	L all outcomes reported	L study appear to be free of other sources of bias	Medium
Garcia-Mata S & Hidalgo-Ovejero A, 2014 [37]	Pulled Elbow (*n* = 115)	Improvement	MT	H not described	H not described	H parents, patients, therapists not blinded	H outcome assessors not blinded	L all expected outcomes reported	L all outcomes reported	L study appear to be free of other sources o bias	Medium
Bek B, et al. 2009 [38]	Pulled Elbow (*n* = 66)	Improvement	MT	H not described	H not described	H no blinding	H outcome assessors not blinded	L intention to treat analysis	L all outcomes reported	L study appears free of other source of bias	Medium
Monaco A, et al. 2008 [39]	Non-Specific Temporomandibular Disorder (*n* = 28)	Improvement	OMT	H not described	H not described	H patients and providers not blinded	H outcome assessor not blinded	H follow up of participants were not discussed	U-PN sample response for each outcome not provided	U-PN small study size	Low
Respiratory											
Guiney P, et al. 2005 [40]	Asthma (*n* = 140)	Improvement	OMT	U-PY not well described “randomization based on a 2:1 ratio”	H not described	H provider not blinded	H outcome assessor not blinded	L all patients accounted for	L all outcomes reported	L study appears free of other sources of bias	Medium
Bronfort G et al. 2001 [41]	Asthma (*n* = 34)	No improvement	CMT	L computer generated	L sealed in opaque envelopes	L blinding of both parents and patients	L outcome assessor blinded	L all patients accounted for	L all outcomes reported	L study appears free of other sources of bias	High
Vandenplas YDE, et al. 2008 [42]	Obstructive Apnea (*n* = 34)	Improvement	OMT	H not described	H not described	L patients blinded	L outcome assessors blinded	H per protocol analysis, 6 participants dropped out and not included in analysis	L all outcomes reported	U-PN small study size, imbalance in sizes of control to study	Medium
Steele D, et al. 2014 [43]	Otitis Media (*n* = 34)	Improvement	OMT	L study used “Research Randomizer”	U-PY randomized tables generated with unique number assignment	H providers not blinded, parents blinded but in treatment room	L outcome assessors blinded	L all patients accounted for	L all outcomes reported	H small sample size, pilot study	Medium
Wahl R, et al. 2008 [44]	Otitis Media (*n* = 90)	No difference	OMT	L randomization in blockes of 8 using random number table	L 2 by2 factorial design	L patients, parents, providers blinded	L outcome assessor blinded	L all patients accounted for	L all outcomes reported	U-PN unequal distribution of risk factors in treatment group	High
Mills M, et al. 2003 [47]	Acute Otitis Media (*n* = 57)	Improvement	OMT	L computer generated	L independent nurse monitored and disclosed by phone	H parents and provider not blinded	L outcome assessor blinded	H per protocol analysis, 19 dropped out and not included in analysis	L all outcomes reported	L study appears free of other sources of bias	High
Special Needs											
Accorsi A, et al. 2014 [48]	Attention-Deficit/Hyperactivity Disorder (*n* = 28)	Improvement	OMT	L permuted-block ratio 1:1 using R statistical program	U- PN allocation was concealed but not described	U -PY patients/parents/providers not blinded but were blinded as to outcomes	L outcome assessors blinded	L all patients accounted for	U -PN adverse events were being collected but not reported	U-PN sample size not justified	High
Khorshid KA, et al. 2006 [50]	Autism (*n* = 14)	Improvement	CMT	H not described	H not described	H patients and providers not blinded	H outcome assessors not blinded	U-PN enrollment number not discussed	L all outcomes reported	U-PN sample size not justified	Low
Wyatt K, et al. 2011 [51]	Cerebral Palsy (*n* = 142)	No improvement	OMT	L telephone based randomization by independent statistician at remote site	L allocation provided by independent statistician at remote site	H parents and patients not blinded	L outcome assessors blinded	L all patients accounted for	L all outcomes reported	U-PN sample size not justified	High
Duncan B, et al. 2008 [53]	Cerebral Palsy (*n* = 55)	Improvement	OMT	L draw technique using stratification	L blinding of concealment	H parents, patients, providers not blinded	L outcome assessor blinded	H per protocol analysis conducted	L all outcomes reported	L study appears free of other sources of bias	High
Duncan B, et al. 2004 [52]	Cerebral Palsy (*n* = 50)	Improvement	OMT	H not described	H not described	H not described	H outcome assessors not discussed	H per protocol analysis conducted	L all outcomes reported	L study appears free of other sources of bias	Low
Raith W, et al. 2016 [54]	Prematurity (*n* = 30)	No difference	OMT/CST	L randomized using block design with block size 6	L sequentially sealed opaque envelopes	L parents and providers blinded	L outcome assessors blinded	L all patients accounted for	L all outcomes reported	L study appears free of other sources of bias	HIgh
Cerritelli F, et al. 2015 [55]	Prematurity (*n* = 695)	Improvement	OMT/CST	L randomized using block design with block size 10	L performed in coordinating center	U-PN providers not blinded	L NICU staff blinded	H per protocol analysis performed	L all outcomes reported	L study appears free of other sources of bias	High
Pizzolorusso G, et al. 2014 [56]	Prematurity (*n* = 110)	Improvement	OMT/CST	L computer generated permuted block	L randomized by IT consultant	U-PN providers not blinded	L outcome assessors blinded	L all patients accounted for	L all outcomes reported	L study appears free of other sources of bias	High
Cerritelli F, et al. 2013 [57]	Prematurity (*n* = 110)	Improvement	OMT/CST	L computer generated permuted block	L random allocation by independent consultant	H parents, patients, providers not blinded	L outcome assessor blinded	H per protocol analysis conducted	L all outcomes reported	L study appears free of other sources of bias	High
Structural											
Cabrera-Martos I, et al. 2016 [58]	Cranial Asymmetry (nonsynostotic plagiocephaly) (*n* = 46)	Improvement	MT/CST	L randomized number generator in blocks of 4	L sealed envelope	H patients and providers not blinded	L outcome assessors blinded	L all outcomes accounted for	L all outcomes reported	L study appears free of other sources of bias	High
Philippi H, et al. 2006 [60]	Postural Asymmetry (*n* = 32)	Improvement	OMT/CST	L block randomization	L sealed in sequentially numbered envelopes	L parents, patients, provider blinded	L outcome assessor blinded	L all outcomes accounted for	L all outcomes reported	L study appears free of other sources of bias	High
Hasler C, et al. 2010 [65]	Scoliosis (*n* = 20)	No improvement	OMT	L block randomization	U-PY consealed envelopes	H patients and provider not blinded	L outcome assessor blinded	L all outcomes accounted for	L all outcomes reported	U-PN small sample size	High
Rowe DE, et al. 2006 [62]	Scoliosis (*n* = 6)	No difference	CMT	L computer generated	L independent personnel provided allocation assignent via e-mail	L patients and provider blinded	L outcome assessors blinded	L all outcomes accounted for	L all outcomes reported	U-PN small sample size	High
Haugen E, et al. 2011 [66]	Torticollis (*n* = 32)	No difference	MT	H not described	U-PY selaed envelope	U-PN patients blinded, providers not blinded	L outcome assessor blinded	U-PN patient description and enrollment not discussed	H not all outcomes reported	U-PN sample size not justified	Medium

Legend: H-High risk of bias; L-Low risk of bias; NA-Not applicable; U-Unclear; PN-Probably No (high risk of bias); PY-Probably Yes (low risk of bias).

Interventions: *CMT* Chiropractic Manipulative Therapy, *CST* Craniosacral Therapy, *MT* Manual Therapy, *OMT* Osteopathic Manipulative Therapy.

**Table 5:** Quality rating of observational studies

Author/year	Study design type	Condition sample size (n)	Result summary	Intervention	Include/exclude	Recruitment strategy	Comparison selection	Blinded outcome assessor(s)	Valid, reliable measures	Length of follow-up	Missing outcomes	Missing harms/ adverse events	Missing confounding variables	Overall quality rating
Gastrointestinal/Urinary														
Tarsuslu T, et al. 2009 [18]	Interrupted time series (with comparison group)	Constipation and Cerebral Palsy (*n* = 13)	No difference	OMT	L does not vary	H not described	H not described	H not blinded	U-PN property measurements not fully evaluated for children	L consistent	L all outcomes discussed	H adverse events not reported	U-PN dietary	Medium
Wiberg K & Wiberg J, 2010 [20]	Interrupted time series (without a comparison group)	Infantile colic (*n* = 749)	No improvement	CMT	NA	NA	NA	H not blinded	U-PN property measurements not fully evaluated for children	H not discussed	L all outcomes discussed	H adverse events not reported	U- PN co-interventions missing	Low
van Poecke A & Cunliffe C, 2009 [25]	Before-after	Nocturnal Enuresis (*n* = 33)	Improvement	CMT	NA	NA	NA	H not blinded	U-PN property measurements not fully evaluated for children	L consistent	L all outcomes discussed	H adverse events not reported	U- PN dietary	Medium
Miller J, et al. 2009 [26]	Before-after	Suboptimal Infant Breastfeeding (*n* = 114)	Improvement	CMT	NA	NA	NA	H not blinded	U-PN property measurements not fully evaluated for children	H not discussed	L all outcomes discussed	L adverse events reported	L confounding variables accounted for	Medium
Vallone S, 2004 [27]	Before-after	Suboptimal Infant Breastfeeding (*n* = 25)	Improvement	CMT/CST	NA	NA	NA	H not blinded	U-PN property measurements not fully evaluated for children	H not discussed	U-PN different outcomes for participants	H adverse events not reported	H no confounding variables included	Low
Musculoskeletal														
Jennings J & Davies G, 2005 [29]	Interrupted time series (without comparison group)	Cuboid Syndrome (*n* = 2)	Improvement	MT	NA	NA	NA	H not blinded	U-PN property measurements not fully evaluated for children	U-PY not different but not specified	L all outcomes discussed	H adverse events not reported	U-PN variables that may influence outcome discussed but no adjustment to outcome taken into account	Medium
Przekop P, et al. 2016 [30]	Before-after	Chronic tension-type headache (*n* = 83)	Improvement	OMT	NA	NA	NA	H not blinded	U-PN property measurements not fully evaluated for children	L consistent	L all outcomes discussed	H adverse events not reported	L confounding variables accounted for	Medium
Marchand A, et al. 2009 [32]	Before-after	Benign infant headache (*n* = 13)	Improvement	CMT	NA	NA	NA	H not blinded	U-PN property measurements not fully evaluated for children	H not discussed	L all outcomes discussed	H adverse events not reported	H medication not accounted for	Low
Walston Z & Yake D, 2016 [34]	Interrupted time series (without comparison)	Mechanical Low Back Pain (*n* = 3)	Improvement	MT	NA	NA	NA	H not blinded	U-PN property measurements not fully evaluated for children	H not consistent	L all outcomes discussed	L adverse events reported	U-PN information not consistently collected	Medium
Hayden J, et al. 2003 [36]	Before-after	Mechanical Low Back Pain (*n* = 54)	Improvement	CMT	NA	NA	NA	H not blinded	U-PN property measurements not fully evaluated for children	L consistent	U-PN not all cases collected	H adverse events not reported	U-PY retrospective data, information not consistently collected	Medium
Respiratory														
Degenhardt B & Kuchera M, 2006 [45]	Before-after	Otitis media (*n* = 8)	Improvement	OMT/CST	NA	NA	NA	H not blinded	U-PN property measurements not fully evaluated for children	L consistent	L all outcomes discussed	H adverse events not reported	U-PN natural course of OM diagnosis, differences in AOM and OM, dietary considerations	Medium
Zhang JQ & Snyder BJ, 2004 [46]	Before-after	Otitis media (*n* = 22)	Improvement	CMT	NA	NA	NA	H not blinded	U-PN property measurements not fully evaluated for children	H not discussed	L all outcomes discussed	L adverse events reported	H several confounding varaibles missing	Medium
Special Needs														
Bramati-Castellarin I, et al. 2016 [49]	Interrupted time series (without comparison)	Autism (*n* = 49)	Improvement	VOMT	NA	NA	NA	H not blinded	U-PN property measurements not fully evaluated for children	L follow up consistent	L all outcomes discussed	H adverse events not reported	U-PY not all confounding variables known	Medium
Structural														
Lessard S, et al. 2011 [59]	Before-after	Cranial asymmetry (nonsynostotic plagiocephaly) (*n* = 12)	Improvement	OMT	NA	NA	NA	L blinded	U-PN property measurements not fully evaluated for children	L follow-up consistent	L all outcomes discussed	H adverse events not reported	U-PN natural course	Medium
Byun S & Han D, 2016 [61]	Before-after	Scoliosis (*n* = 5)	Improvement	CMT	NA	NA	NA	H not blinded	L Cobb angle	L follow-up consistent	L all outcomes discussed	H adverse events not discussed	H confounding variables not accounted for, no mention of natural course	Medium
Morningstar M, et al. 2004 [63]	Before-after	Scoliosis (*n* = 6)	Improvement	CMT	NA	NA	NA	H not blinded	L Cobb angle	H length of follow-up similar but some patients had received prior treatment	L all outcomes discussed	H adverse events not reported	H confounding variables not accounted for, no mention of natural course	Low
Lantz C & Chen J, 2001 [64]	Before-after	Scoliosis (*n* = 42)	No improvement	CMT	NA	NA	NA	L blinded	L Cobb angle	H follow-up not consistent	L all outcomes discussed	H adverse events not reported	H confounding variables missing, no mention of natural course	Medium
Saedt E, et al. 2018 [67]	Before-after	Upper cervical dysfunction (*n* = 307)	Improvement	MT	NA	NA	NA	L blinded	U-PN property measurements not fully evaluated for children	L follow-up consistent	L all outcomes discussed	L adverse events discussed	U-PY not all confounding variables known	High

Legend: H-High risk of bias; L-Low risk of bias; NA-Not applicable; U-Unclear; PN-Probably No (high risk of bias); PY-Probably Yes (low risk of bias)

Interventions: *CMT* Chiropractic Manipulative Therapy, *CST* Craniosacral Therapy, *MT* Manual Therapy, *OMT* Osteopathic Manipulative Therapy

Overall, we found 23 studies that used OMT (7 of which specifically used cranial therapies and 1 VOMT); 17 studies used CMT/SMT (including one using Toftness technique, one using an upper cervical technique, one using a neuroimpulse instrument, and one using cranial therapy with CMT), 10 studies used mobilizations (1 also using CST).

#### Pediatric clinical conditions

1. Gastrointestinal/urinary conditions

Table 6 provides a summary of the 10 studies that investigated the clinical effects of manual therapy for conditions categorized as “gastrointestinal/urinary conditions”. One of the studies investigated the use of manual therapy for constipation [18], five for infantile colic [19–23], one for children with dysfunctional voiding [24], one for pediatric nocturnal enuresis [25], and two studies for suboptimal infant breastfeeding [26, 27]*.*

1.1. Constipation

**Table 6:** Data extraction for the gastrointestinal/urinary studies

Condition	Author/year	Study objective	Study design Sample size Intervention	Patient description/condition	Primary/ main outcome(s)	Main results/ conclusions	Adverse events
Constipation	Tarsuslu T, et al. 2009 [18]	Investigate potential effects of osteopathic treatment on constipation in children with cerebral palsy.	Interrupted Time Series (with comparison group) *n* = 13 OMT	Children with CP, ages 2–16, with constipation	Defecation frequency, gross motor function classification system, Modified Ashworth scale, functional independence measure for children, constipation assessment scale, visual analog scale	Both groups showed significant changes from all baseline measures at 3 mos.	There was no mention of adverse events made in this study.
Infantile Colic	Miller JE, et al. 2012 [19]	Two-fold: 1. Determine efficacy of chiropractic manipulation therapy for infants with colic; and 2. Parental reporting bias.	RCT *n* = 104 CMT	Infants < 8 weeks, diagnosed with colic	Decreased crying (as assessed by parent questionnaire and 24 h crying diary)	1. Greater decrease in crying in colicky infants treated with CMT compared to infants who were not treated. 2. Unlikely that observed treatment effect is due to bias on part of reporting parent.	One patient in the control group noted increased crying.
	Wiberg K & Wiberg J, 2010 [20]	Investigate if the outcome of excessively crying infants treated with chiropractic manipulation is associated with age.	Interrupted Time Series (without comparison group) *n* = 749 CMT	Healthy, thriving infants, ages 0–3 months, who fit diagnostic criteria of infantile colic	Parent report of crying: classified as “improved”, “uncertain recovery”, “non recovered”	No apparent link between clinical effect of chiropractic treatment and a natural crying pattern was found, Slightly older age was found to be linked to crying infants with clinical improvement	There was no mention of adverse events made in this study.
	Browning M & Miller J, 2008 [21]	To compare chiropractic manual therapy and occipital-sacral decompression in the treatment of infant colic.	RCT *n* = 43 CMT	Infants < 8 weeks, who cried more than 3 h a day for at least 4 of the previous 7 days	Change in group mean daily hours of crying (recorded in crying diary)	Mean hours of crying were significantly reduced in both groups. Both treatments appear to offer benefits to infants with colic. There was no difference between two treatment approaches.	There was no mention of adverse events made in this study.
	Olafsdottir E, et al. 2001 [22]	To evaluate chiropractic spinal manipulation management on infantile colic.	RCT *n* = 86 CMT	Infants ages 3–9 weeks, diagnosed with infantile colic	24 h diary of infant’s crying (crying diary) completed by parent; Parent report of effect after last visit (8–14 days later)	No difference between groups with either outcome.	There was no mention of adverse events made in this study.
	Hayden C & Mullinger B, 2006 [23]	To determine the impact of cranial osteopathy on infantile colic.	RCT *n* = 28 OMT/CST	Infants 1–12 weeks, with signs of infantile colic that included; 90 min/24 h. of inconsolable crying on 5 out of 7 days and additional clinical signs such as borborygmi, knees drawn up to chest, fists clenched, backward bending of head or trunk	Parents record of time spent crying and sleeping in a 24-h diary	No between group comparisons done. While both groups, demonstrated decreases, only the OMT/CST group had significant reduction for time spent crying and sleeping.	There was no mention of adverse events made in this study.
Pediatric dysfunctional voiding	Nemett D, et al. 2008 [24]	To determine whether manual physical therapy-osteopathic approach added to standard treatment improves dysfunctional voiding more effectively than standard treatment alone.	RCT *n* = 21 OMT	Children ages 4–11, diagnosed with dysfunctional voiding and symptoms of daytime incontinence and or vesicoureteral reflux	Improved dysfunctional voiding symptoms; 1. improved or resolved vesicoureteral reflux 2. elimination of post-void urine residuals	Results suggest that manual physical therapy-osteopathic approach treatment can improve short-term outcomes in children with dysfunctional voiding, beyond improvements observed with standard treatments.	There was no mention of adverse events made in this study.
Nocturnal Enuresis	van Poecke A & Cunliffe C, 2009 [25]	To evaluate the effect of chiropractic treatment on the wet night frequency of patients with nocturnal enuresis.	Before-After *n* = 33 CMT	Children ages 3–18, diagnosis of nocturnal enuresis	Diary of wet night frequency, diurnal urinary output	66.6% resolution rate within 1 year, indication for possible effectiveness of chiropractic treatment (Neuroimpulse instrument) in patients with primary nocturnal enuresis.	There was no mention of adverse events made in this study.
Suboptimal infant breastfeeding	Miller J, et al. 2009 [26]	To determine the effect of chiropractic manipulative therapy on infants who had difficulty breastfeeding.	Before-After *n* = 114 CMT	Infants ages 2 days - 12 weeks diagnosed by medical provider with feeding difficulties	Mother’s report of exclusivity of breastfeeding, rating of improving and infant weight gain	Exclusively of breastfeeding was accomplished in 78%.	No negative side effects were reported.
	Vallone S, 2004 [27]	To investigate problems interfering with successful breastfeeding and to see if proper lactation management can increase the bonding experience.	Before-After *n* = 25 CMT/CST	Infants ages 5 days - 12 weeks, referred by other healthcare providers as having difficulty breastfeeding	Improvement in ability to latch and ability to breastfeed	> 80% of infants experienced improvement in latch and ability to breastfeed.	There was no mention of adverse events made in this study.

One study was found that investigated the use of OMT for constipation [18].

Tarsuslu et al. conducted a medium quality, interrupted time-series with a comparison group that investigated the potential effects of OMT on constipation in 13 children ages 2–16 with cerebral palsy. The children were put into one of two groups with no description of how this allocation happened. The first group received OMT alone and the second group received OMT in addition to medical treatment. Both groups showed significant changes from all baseline measures at 3 months. The baseline measures included defecation frequency, gross motor function, and functional independence measure. Group 1 showed significantly favorable changes in defecation frequency and constipation scale at 6 months. Group 2 showed significantly favorable changes from baseline measures at 6 months. The researchers suggest advanced additional studies should be conducted. There was no mention of adverse events made in this study [18].

*Overall Summary:*

***Inconclusive (unclear)*** *for use of OMT in treating constipation.*

1.2. Infantile colic

Four of the five studies investigated the use of CMT in treating infantile colic; three of these studies were high quality RCTs [19, 21, 22] and one low quality retrospective investigation of the clinical records [20]. One medium quality prospective RCT investigated the use of OMT cranial therapy [23].

A high quality parent-blinded RCT, authored by Miller et al., showed favorable results in treating 104 colicky infants less than 8 weeks of age with CMT. This study had two objectives; the first was to determine the efficacy in treating colic with CMT and the second was to determine if parental reporting bias contributes to the success of the treatment. The infants were randomized into 3 groups: infant treated-parent aware; infant treated-parent unaware; and infant not treated-parent unaware. The outcomes were determined by a decrease in crying time, as assessed by a parent questionnaire and a 24 h crying diary. The study found there was a greater decrease in crying time in the infants treated with CMT, either parent aware or unaware, compared to infants who were not treated, concluding that parents did not appear to contribute to the observed treatment effects in the study. Adverse event was reported in one patient in the control (non-treatment) group that reported increased crying [19].

Wiberg et al. conducted a low quality interrupted time series without a comparison group observational study that looked at 749 clinical records of infants 0–3 years of age who fulfilled the study’s definition of excessive crying. This study investigated if the outcome of excessively crying infants treated with CMT was associated with or partially associated with age in the natural decline in crying with age in infants. The outcomes were determined by the parents recording crying in the infants as “improved”, “uncertain”, or “non-recovery”. These researchers concluded that there was no apparent link between the clinical effect of chiropractic treatment and improvement in the crying patterns. Limitation of the study was that it was pragmatic, thus not standardized on management or CMT technique. There was no mention of adverse events in this study [20].

Browning and Miller conducted a high quality parent-blind RCT involving 43 infants less than 8 weeks of age that presented with infantile colic. The study objective was to compare two intervention groups in the treatment of infantile colic. One intervention group received CMT and the other occipital-sacral decompression. The outcomes were determined by the change in mean daily hours of crying as recorded in a crying diary by a parent. Although the mean hours of daily crying were statistically significantly reduced in both study groups, there were no statistically significant differences between them. The researchers noted that although all participants’ symptoms improved prior to the normal remission age of colic, the natural course of remission could not be ruled out. There was no mention of adverse events made in this study [21].

Olafsdottir et al. conducted the third high quality RCT that set out to evaluate the effect of CMT on infantile colic. This study included 86 colicky infants (46 receiving CMT, 40 in control group) at 3–9 weeks of age. The outcome was determined by the parents recording the hours of infant crying per 24 h period in a crying diary. The results showed no statistically significant improvement in the infants in either group. There was no mention of adverse events made in this study [22].

Another medium quality prospective, open-controlled RCT that investigated the impact of cranial osteopathy on infantile colic in 28 infants was conducted by Hayden et al. These researchers found a reduction in crying times (63%), improved sleeping (11%), and a need for less parental attention. Due to the favorable findings of this study, the researchers suggested that a larger scale study is warranted. There was no mention of adverse events made in this study [23].

*Overall Summary:*

***Inconclusive (unclear)*** *for CMT in treating infantile colic.*

***Inconclusive (favorable)*** *for OMT/CST in treating infantile colic.*

1.3. Pediatric dysfunctional voiding

A medium quality RCT sought to determine whether OMT in addition to standard treatment improved dysfunctional voiding in 21 children diagnosed with pediatric dysfunctional voiding. Improvements in short-term outcomes in children with dysfunctional voiding were reported beyond improvements observed with standard treatment. No mention of adverse events were reported in this study [24].

*Overall Summary:*

***Inconclusive (favorable)*** *evidence for use of OMT plus standard treatment to improve dysfunctional voiding.*

1.4. Pediatric nocturnal enuresis

A medium quality before-after retrospective record review of 33 consecutive patients over a three-year period found somewhat favorable results using CMT, specifically utilizing the Neuroimpulse protocol. The children were between the ages 3–18 with primary nocturnal enuresis. The frequency of wet nights was abstracted from the records at 3, 6, 9 and 12 months after the commencement of treatment. The records found 22 patients showed complete resolution of primary nocturnal enuresis during the 12 months after commencement of chiropractic care. The resolution rate was 66.6% within 1 year with the mean number of treatments in the responder’s group being 2.05 ± 1.33. There was no mention of adverse events made in this study [25].

*Overall Summary:*

***Inconclusive (unclear)*** *evidence for use of CMT to improve nocturnal enuresis.*

1.5. Suboptimal infant breastfeeding (SIB)

Two case series with pre and post measurements investigated the use of CMT on infants with SIB [26, 27].

A medium quality before-after case series investigated the effect of CMT on 114 infants with SIB*,* 112 classified with an ineffective suck (grades 0–2) and 2 having excessive suck (grade 4) as objectively determined with a suck grading chart. The results of this study showed favorable improvement in all the infants after four treatments (78% were able to exclusively breastfeed). Outcomes included the mother’s report of improved weight gain and a specific list of historical data and clinical examination findings including improvements in suck reflex grading. No negative side effects were reported [26].

A low quality before-after case series of 25 infants with SIB set out to determine if proper lactation might increase the bonding experience between mother and infant following CMT/CST. This study reported improvement in the ability to latch after the infants received CMT (which included craniosacral treatment). The study’s authors posited CMT/CST in the early stages of neurological imprinting appear to safely and effectively address the craniocervical dysfunction and help restore natural efficient sucking patterns for infants who are unable to latch. There was no mention of adverse events made in this study [27].

*Overall Summary:*

***Inconclusive (favorable)*** *evidence for use of CMT/CST for children with* SIB*.*

2. Musculoskeletal conditions

Table 7 summaries the 12 studies that investigated the clinical effects of manual therapy for conditions categorized as “musculoskeletal conditions”. One of these investigated the use of manual therapy on clubfoot [28] and one on cuboid syndrome [29]. Three of these studies investigated the use of manual therapy for headaches [30–32], four for low back pain [33–36], two investigated pulled elbow [37, 38], and one study for temporomandibular (TMD) dysfunction [39].

2.1. Clubfoot

**Table 7:** Data extraction for the musculoskeletal studies

Condition	Author/ year	Study objective	Study design sample size intervention	Patient description/condition	Primary/main outcome(s)	Main results/conclusions	Adverse events
Clubfoot	Nilgun B, et al. 2011 [28]	To determine efficacy of physical therapy, including manual mobilization, as adjunct to Ponseti technique in idiopathic clubfoot.	RCT *n* = 29 MT	Children ages 3 and under, Dimeglio Score of 17 or under with idiopathic clubfoot	Improvements in passive ranges of motion for plantar flexion, inversion, eversion, rear foot varus angle and forefoot adduction angle and decrease in Dimeglio Score	Treatment procured a statistically significant improvements in ranges of motion, Dimeglio Score and decrease of rear foot varus angle in the study group.	There is no mention of adverse events made in this study.
Cuboid Syndrome	Jennings J & Davies D, 2005 [29]	Describe the examination and treatment of the cuboid syndrome following lateral ankle sprain.	Interrupted Time Series (with comparison group) *n* = 2 MT	7 patients age range 15–36 (2 pediatric patients can be isolated), with cuboid syndrome	Visual Analog Scale: Pre- and post-treatment	All patients had substantial resolution of symptoms following cuboid manipulation.	There is no mention of adverse events made in this study.
Headache	Przekop P, et al. 2016 [30]	Evaluate and compare a multimodal with pharmacologic treatment for the prevention of chronic tension type headaches (CTTH) in adolescents.	Before-After *n* = 83 OMT	Children ages 13–18, diagnosed with CTTH	5 main effects: headache frequency, pain intensity, general health, pain restriction and number of tender points	Both approaches showed significant improvements across all 5 main effects outcomes, but multimodal treatments produced more favorable results in headache frequency, general health, and number of tender points.	There is no mention of adverse events made in this study.
	Borusiak P, et al. 2010 [31]	To investigate the efficacy of spinal manipulative therapy in adolescents with recurrent headache.	RCT n = 52 MT	Adolescents ages 7–15, with cervicogenic headache	Assessment of; percentage of days with headache, total duration of headache, days with school absence due to headache, consumption of analgesics, and intensity of headache	No difference in any outcome measure between placebo and cervical spine manipulation.	No serious or moderate adverse events were noted. Minor adverse events occurred in both groups that included; hot skin in 15 patients (treatment group 6, placebo 9), dizziness in 11 patients (treatment group 7, placebo 4). There was reported transitory increase in headache intensity and frequency being reported for up to 4 days (treatment group 8, placebo 6).
	Marchand A, et al. 2009 [32]	To conduct a retrospective file search of infants presenting with probable benign infantile headache at a chiropractic teaching clinic.	Before-After *n* = 13 CMT	Children ages 2 days - 8.5 months, with benign infant headache	Reduction in behavioral findings recorded verbatim by parents such as; grabbing holding face, ineffective latching, grimacing and positional discomfort, rapping head against floor, photophobia, and anorexia.	All 13 consecutive cases had favorable results based on parent report of outcomes.	There is no mention of adverse events made in this study.
Low Back Pain	Evans R, et al. 2018 [33]	To compare 12 weeks of chiropractic manipulative therapy combined with exercise therapy to exercise therapy alone in the treatment of chronic lower back pain in children.	RCT *n* = 185 CMT	Children ages 12–18, with chronic lower back pain	Primary outcome - self-reported level of low back pain (11 box numerical rating scale), Secondary outcomes - patient-rated disability (18 item Roland-Morris Disability questionnaire), quality of life (23 item PedsQL), improvement (9-point scale), frequency of medication use for low back pain (days/week), patient satisfaction with care (7-point scale)	Chiropractic manipulative therapy plus exercise resulted in larger reduction in primary outcome of pain severity over the course of 1 year.	Side effects were similar in both groups, mild and self-limiting and occurred at a frequency comparable to adult population.
	Walston Z & Yake D, 2016 [34]	To illustrate the feasibility and safety of lumbar manipulation as an adjunct to exercise for treatment of adolescent population with mechanical low back pain.	Interrupted Time Series (without comparison group) *n* = 3 MT	Adolescents ages 13–15, with mechanical low back pain	Pain measured on numerical pain rating scale and disability (Oswestry) for each patient	All outcome showed improvements (0/10 on numeric scale and 0% in the Oswestry disability index) for each patient.	No adverse reactions were reported or observed with any episode of manipulation.
	Selhorst M & Selhorst B, 2015 [35]	To assess efficacy of lumbar manipulation in addition to a 4-week physical therapy exercise program.	RCT *n* = 35 MT	Adolescents ages 13–17, with mechanical low back pain of < 90 days	Patient Specific Functional Scale, pain (11-point Numerical Pain Rating Scale), and Global Rating of Chance scales	No difference between groups for Patient Specific Functional Scale, pain, or Global Rating of Chance scales. All patients improved.	Two patients in both the sham and manipulation group had an adverse reaction at 1 week. No patients in either groups reported adverse reactions at either 4 weeks or 6 months. They concluded that no additional risk of having an adverse reaction were noted in this study.
	Hayden J, et al. 2003 [36]	To describe chiropractic management, outcomes, and factors associated with outcomes for low back pain in childhood.	Before-After *n* = 54 CMT	Children ages 4–18, with acute mechanical low back pain	Subjective assessment of improvement on a 5-point rating scale (Pediatric Visual Analog Scale)	Over a course of 4–6 weeks of chiropractic management, 55–62% of patients had improvement that met the study’s stringent criteria and 82–87% had much improvement.	Complications from chiropractic patient management were collected with none noted throughout the study data collection period.
Pulled Elbow	García-Mata S & Hidalgo-Ovejero A, 2014 [37]	To determine the relative efficacy of two pulled elbow reduction maneuvers, hyper pronation and supination-flexion.	RCT *n* = 115 MT	Children ages 1–5, with pulled elbow	Reduction of pulled elbow verified by observing active flexion and extension	Both maneuvers were effective with a higher first-attempt success rate with hyper pronation.	There is no mention of adverse events collected in this study.
	Bek D, et al. 2009 [38]	To compare the reduction efficiency of hyper pronation to supination-flexion maneuvers for a pulled elbow.	RCT *n* = 66 MT	Children ages 1–5, with pulled elbow	Reduction of pulled elbow indicated by child using the arm	Final reduction rates similar. Hyper pronation maneuver was more successful on the first attempt.	There is no mention of adverse events collected in this study.
Temporomandibular Disorder	Monaco A et al. 2008 [39]	To evaluate the effects of osteopathic manipulative therapy on mandibular kinematics in patients with temporomandibular dysfunction.	RCT *n* = 28 OMT	Children average age 12, diagnosed with TMD	Objective measures pre- and post-treatment using kinesiographic tracings to assess mandibular movement	Osteopathic manipulation made significant improvements in maximal mouth opening and in maximal mouth opening velocity.	There is no mention of adverse events made in this study.

One study was found that investigated the use of MT on patients with clubfoot [28].

A low quality RCT conducted by Nilgun et al. investigated the effectiveness of intensive physical therapy (including mobilization technique) as an adjunct to Ponseti technique in 29 children (average age 15–12 months) with idiopathic clubfoot. Using the Dimeglio classification system they reported a statistically significant improvement in the group that received both MT and the Ponseti technique combined. The study group received the intervention once per day, 5 days a week for 1 month. There is no mention of adverse events made in this study [28].

*Overall Summary:*

***Inconclusive (favorable)*** *evidence for the use of MT combined with Ponseti technique in children with clubfoot.*

2.2. Cuboid syndrome

One study was found that investigated the use of MT on patients with cuboid syndrome [29].

A medium quality interrupted time-series without a comparison group described the proper examination, evaluation, and treatment of cuboid syndrome with manual manipulation following lateral ankle sprains in 7 patients aged 15–36 of which 2 children met our inclusion (ages 15 and 16). Using visual analog scales pre and post treatment Jennings et al. reported patients’ subjective pain at rest, during palpation, during midtarsal mobility testing, with gait, and with single-leg hop. Both children were diagnosed with this condition and received a cuboid manipulation. They each required only one treatment and were able to return to competitive activity with one treatment without injury recurrence. There is no mention of adverse events made in this study [29].

*Overall Summary:*

***Inconclusive (unclear)*** *evidence for MT in patients with cuboid syndrome.*

2.3. Headache

Three studies investigated the use of manual therapy on pediatric headaches. One medium quality before-after study investigated the use of OMT on chronic tension-type headaches in adolescents [30]. One medium quality RCT that was stopped early (before recruitment goal based on interim analysis) evaluated the clinical effectiveness of MT [31]. One low quality retrospective case series with pre and post measurements looked at the CMT [32].

Przekop et al. conducted a medium quality before-after observational study that compared multimodal (OMT) and pharmacologic effects on chronic tension-type headaches (CTTH). This study included 83 patients, (67 females and 16 males), between the ages of 13 and 18. Outcome measures included: headache frequency, pain intensity, general health, pain restriction and the number of tender points as found by the provider. They reported that both multimodal and pharmacologic treatments were effective for CTTH; however, results from multimodal treatment produced more favorable results in headache frequency, general health and in the number of tender points elicited. There was no mention of adverse events in this study [30].

Borusiak et al. conducted a medium quality RCT comparing the use of cervical MT to a sham MT in 56 children with cervicogenic headaches. Of these, data sets of 52 children were analyzed (mean age 11.6 years). Outcomes included: percentage of days with a headache, total duration of headache in hours, percentage of days missing school, percentage of days with necessity of analgesic medication, and intensity of headache based on a 10-point numerical analog scale. No significant difference was reported for any outcome measure. They did note that baseline and follow-up frequency of days with headache was reduced in both groups however, the differences were not significant. Minor adverse events occurred in both groups with no serious or moderate adverse events reported [31].

Marchand et al. conducted a low quality before-after case series that investigated the effects CMT for 13 infants (aged 2 days to 8.5 months) with probable benign infant headache. Outcome measures were changes noted in behavioral findings as reported verbatim by parents including: less grabbing or holding of the face, improved latching, less grimacing and positional discomfort, less rapping of the head against the floor and less photophobia and anorexia. They reported that all of the patients responded favorably to CMT and that a therapeutic trial is warranted. There is no mention of any adverse events in this article [32].

*Overall Summary:*

***Inconclusive (unclear)*** *for the use of OMT for chronic tension-type headaches in adolescents, for the use of MT for cervicogenic HA, and for the use of CMT for benign infant headache.*

2.4. Low back pain (LBP)

Four studies investigated the use of manual therapy for LBP in the pediatric population. Two studies looked at the use of CMT; one high quality RCT, the other a medium quality before-after study [33, 36]. The other two looked at the use of MT; a medium quality interrupted time-series, the other a medium quality RCT [34, 35].

Evans et al. presented a high quality RCT with a comparison group between CMT with exercise against solely focusing on exercise therapy. The patients included a range of ages between 12 and 18 years, concluding with 185 total patients. They concluded that adolescents showed that by adding CMT with exercise therapy, resulted in a larger reduction in the primary outcome (visual analog scale) of pain severity over the course of 1 year. The study reported minor self-limiting adverse events that were about equal frequency in both groups [33].

Walston and Yake conducted a medium quality interrupted time- series without a comparison group of 3 patients (age range 13 through 15). They showed feasibility and safety of lumbar manipulation with exercise in the adolescent population with LBP. Patient centered outcomes used included: subjective pain measured on a numeric pain rating scale and the use of Oswestry disability index. All outcomes showed improvement for all patients with no adverse reactions to manipulation [34].

The medium quality RCT of 35 patients (age range 13–17, mean 14.9 years) with mechanical LBP of less than 90 days, was conducted to evaluate the clinical effects of MT in addition to an exercise program. Eighteen children received MT and 17 received a sham manipulation, which consisted of the child lying on their side and a therapist passively flexing both hips until slight lumbar flexion. Patient centered outcomes utilized included, Patient Specific Functional Scale and Numerical Pain Rating Scale. Global Rating of Change scales was used to evaluate perceived improvement. Both groups of patients reported improvements in LBP. The authors reported that there was no additional risk for lumbar manipulation, as both groups reported the same number of adverse events [35].

Hayden et al. conducted a medium quality before- after cohort study without a control group that investigated the effectiveness of CMT for LBP for 54 patients ranging in age between 4 and 18 years. They reported that the majority of the patients responded favorably and there were no reported adverse events. The researchers were quick to point out that a causal relationship between CMT and improvements in pediatric LBP could not be established due to both the small study size and the observational design of the study itself. Complications from chiropractic patient management were collected with none noted throughout the study data collection period [36].

*Overall Summary:*

***Moderate (positive)*** *evidence for the use of CMT for adolescent LBP.*

***Inconclusive (unclear)*** *evidence for the use of MT for pediatric mechanical LBP.*

2.5. Pulled (nurse’s) elbow

Two RCTs met our inclusion criteria and investigated the effectiveness of two MT maneuvers for the reduction of pulled elbow. It is important to point out that both of these studies compared two different types of manipulation and both show favorable results on pulled elbow [37, 38].

A medium quality RCT of 115 patients (mean age 2.3 years old) was conducted by Garcia-Mata et al. and sought to determine which procedure was the most effective to reduce a pulled elbow. There were 65 patients allocated to the hyper pronation group and 50 in the supination-flexion group. The hyper pronation group was found to be more efficient on reduction at the first attempt. There is no mention of adverse events made in this study [37].

A medium quality RCT compared the efficiency of hyper pronation and supination flexion maneuvers in the reduction of pulled elbow on 66 children (34 hyper pronation-flexion and 32 supination-flexion) with an average age of 28 months. Successful reduction was considered by the observation of the child being able to use the arm after the reduction. Although the authors concluded that final reduction rates were similar in both groups they found that the hyper pronation maneuver was more efficient on the first attempt. There is no mention of adverse events made in this study [38].

*Overall Summary:*

***Moderate (positive)*** evidence for use of CMT/CST for children with SIB.

2.6. Temporomandibular dysfunction (TMD)

One study was found that investigated the use of OMT for TMD dysfunction [39].

A low quality RCT conducted by Monaco et al. evaluated the effects of OMT on mandibular kinematics in 28 children diagnosed with non-specific temporomandibular disorders. Kinesiographic tracings using K71 measured mandibular incisor-point movement in three dimensions was the only outcome assessed. The results of this study showed a significantly statistical improvement in the maximal mouth opening velocity in the study group. It was reported that the use of OMT improved non-specific TMD. There is no mention of adverse events made in this study [39].

*Overall Summary:*

***Inconclusive (favorable)*** *evidence for OMT in pediatric* TMD.

3. Respiratory and eyes, ears, nose, and throat (EENT) conditions

Table 8 summarizes the eight studies that investigated respiratory, EENT conditions. In total, there were two studies that investigated children with asthma [40, 41], one study that investigated children with obstructive apnea [42], and five studies investigated children with otitis media [43–47].

3.1. Asthma

**Table 8:** Data extraction for respiratory studies

Condition	Author/year	Study objective	Study design sample size intervention	Patient description/ condition	Primary/main outcome(s)	Main results/conclusions	Adverse events
Asthma	Guiney P, et al. 2005 [40]	To demonstrate the therapeutic relevance of osteopathic manipulation in the pediatric asthma population.	RCT *n* = 140 OMT	Children ages 5–17, diagnosed with asthma by guidelines from NIH	Peak Expiratory Flow Rates	There was statistically significant improvement of 7 L per minute to 9 L per minute for peak expiratory flow rates in the treatment group.	There was no mention of adverse events made in this study.
	Bronfort G, et al. 2001 [41]	To determine if chiropractic manipulative therapy in addition to optimal medical management resulted in clinically important changes in asthma-related outcomes.	RCT *n* = 34 CMT	Children ages 6–17, with persistent asthma	Pulmonary function tests, diary recording peak expiratory flow and inhaler use, questionnaires assessing quality of life, asthma severity and improvement	Little to no change in pulmonary function tests at 12 weeks and no change in patient, parent/guardian or pulmonologist rated improvement	There was no mention of adverse events made in this study.
Obstructive Apnea	Vandenplas Y, et al. 2008 [42]	To evaluate if osteopathy can influence the incidence of obstructive apnea during sleep in infants.	RCT *n* = 34 OMT	Infants aged 1.5–4 months, with obstructive apnea as determined by a polysomnographic test	Decrease in the number of obstructive apneas as measured by polysomnography.	Infants aged 1.5–4 months, with obstructive apnea as determined by polysomnographic	There was no mention of adverse events made in this study.
Otitis Media	Steele D, et al.2014 [43]	To evaluate the efficacy of an osteopathic manipulative treatment protocol on middle ear effusion resolution following acute otitis media.	RCT *n* = 52 OMT	Infant ages 6–24 months, with acute otitis media and abnormal tomogram	Tympanometer and acoustic reflectometer	Both tympanometer data and acoustic reflectometer analysis demonstrated significantly significant improvement in middle ear effusion at visit 3 in the standard care plus osteopathic treatment group.	There were no serious adverse events reported during the study.
	Wahl R, et al. 2008 [44]	To assess the efficacy of Echinacea and osteopathic manipulative treatment for preventing acute otitis media.	RCT *n* = 90 OMT	Children aged 12–60 months, with recurrent otitis media	Reduction in future episodes of OM	No interaction was found between Echinacea and osteopathic manipulation. Echinacea was associated with a borderline increased risk of having at least one episode of acute otitis media during 6-month follow-up compared to placebo. Osteopathic manipulation did not significantly affect risk compared to sham.	“One subject withdrew from the study following an adverse effect (vomiting after taking the Echinacea placebo). One additional subject reported adverse effects (vomiting and non-urticarial rash 2 days after starting Echinacea for a viral upper respiratory illness) but did not withdraw. Neither adverse effect was considered to have been caused by the study medication.
	Degenhardt B & Kuchera M, 2006 [45]	Does osteopathic manipulation decrease the recurrence of otitis media?	Before-After *n* = 8 OMT/CST	Infants ages 7–35 months, with recurrent otitis media	Decreased incidence of acute otitis media	5 participants had no recurrence after 1 year follow-up. 1 participant had 1 recurrence. 2 participants had a short-term of no recurrence only.	There is no mention of adverse events made in this study.
	Zhang J & Snyder B, 2004 [46]	To study the effect of Toftness chiropractic adjustment for acute otitis media.	Before-After *n* = 22 CMT	Children ages 9 months −9 years, with acute otitis media	Tympanic Membrane visualization via otoscopic exam and oral temperature	After Toftness chiropractic adjustment, red and bulging tympanic membrane returned to normal in 95% of children. A decrease in average oral temperature was noted.	“During the study protocol, no side effects or deterioration of clinical presentations were found among 21 children with otitis media.”
	Mills M, et al. 2003 [47]	To evaluate the effect of usual care and osteopathic manipulation for children with acute otitis media.	RCT *n* = 57 OMT	Children ages 6 months - 6 years, with recurrent otitis media	Decreased frequency of acute otitis media, antibiotic us, surgical interventions, and improved tympanometric and audiometric performance	Intervention group had fewer episodes of acute otitis media, fewer surgical procedures and an increased frequency of more normal tympanogram readings.	There were no adverse events reported during the study.

Two studies were identified that investigated the use of manual therapy for the treatment of pediatric asthma. One study was a medium quality and investigated OMT [40]. The other study was a high quality pilot RCT and investigated CMT [41].

Guiney et al. conducted a medium quality RCT and reported favorable results with the use of OMT in 140 patients (90 treatment group, 50 control group), ages 5–17 with asthma. The primary outcome was improved peak expiratory flow rates. Their results show a statistically significant improvement from 7 L/min to 9 L/min for peak expiratory flow rates. No mention of adverse events was noted in this study [40].

Bronfort et al. conducted a high quality pilot RCT that investigated if CMT in addition to medical management would result in clinically important changes in asthma-related outcomes. This study included an observation component, but no actual data was available to include in this review. Their study included 34 children aged 6–17 years of age with persistent asthma. The main outcomes were determined by pulmonary technicians at baseline and at 12 weeks. They looked at diaries of recording peak expiratory flow and inhaler use, questionnaires assessing quality of life, asthma severity, and improvements. They found little to no change in pulmonary function tests at 12 weeks and no change in patient or pulmonologist rated improvement with the use of CMT. However, Bronfort et al. did report improvement in patient-centered outcomes such as quality of life, even 1 year after the last treatment. No mention of adverse events was noted in this study [41].

*Overall Summary:*

***Inconclusive (favorable)*** *for OMT in treating asthma.*

***Inconclusive (unclear)*** *for CMT in treating asthma.*

3.2. Obstructive apnea

One study was found that investigated the use of OMT on obstructive apnea [42].

A medium quality pilot RCT by Vandenplas et al. sought to investigate if OMT can influence the incidence of obstructive apnea during sleep in infants. This study of 34 infants, ages 1.5–4 months diagnosed with obstructive apnea showed a significant decrease in the number of observed apnea episodes in the OMT group compared to the control group. The main outcome measured was a decrease in the incidence of apnea with the suggestion for additional research. No mention of adverse events was noted in this study [42].

*Overall Summary:*

***Inconclusive (favorable)*** *evidence for OMT in treating obstructive apnea.*

3.3. Otitis media

Five studies investigated the clinical effectiveness of manual therapy on otitis media that met our inclusion criteria. Four of the studies investigated the use of OMT. Of these, two were of high quality and two were of medium quality [43–45, 47]. One medium quality study looked at the use of CMT (specifically Toftness technique) for acute otitis media [46]. All but one of the OMT studies showed favorable results on the use of MT for acute otitis media.

Steele et al. conducted a medium quality prospective, pilot RCT (stopped before it reached its recruitment goal of 80 patients) that evaluated 52 infants ages 6–24 months with acute otitis media and abnormal tomograms. The primary outcome was measured with a tympanometer and an acoustic reflectometer. They determined there was faster resolution in middle ear effusion in 2 weeks with what they described as “standardized OMT”. There were no serious adverse events reported during this study [43].

A high quality RCT evaluated the use of *Echinacea purpurea* and OMT on 90 (84 completed the study) infants aged 12–60 months with recurrent otitis media. The main outcome of the study was a reduction in the incidence of recurrent otitis media. As reported in monthly telephone interviews and at the 3- and 6-month visits, there was no statistically significant difference in reporting of any side effects between placebo and treatment groups for either echinacea or OMT. One participant withdrew from the study following adverse events (vomiting after taking the echinacea placebo). One additional participant reported adverse events (vomiting and non-urticarial rash 2 days after starting echinacea for a viral upper respiratory illness) but did not withdraw [44].

A medium quality before-after cohort, practice based study evaluated 8 infants ages 7–35 months with recurrent acute otitis media was undertaken by Degenhardt et al. The main outcome was a decreased incidence of otitis media. The results of this study were that 5 of the 8 children had no recurrence after 1 year follow up, one had 1 recurrence, and 2 of the 8 children had a short period of no recurrence after receiving OMT. In the method section of this study, the OMT used met the description of craniosacral therapy (CST). It is also important to note that all participants in this study were also under concurrent medical care. No mention of adverse events was noted in this study [45].

A medium quality study before-after case series investigated 22 children ages 9 months to 9 years with acute otitis media showed favorable results utilizing Toftness chiropractic technique, a type of low force technique chiropractic system. The primary outcome measures utilized in the study was otoscopic visualization and oral temperature. The researchers of this study state that otitis media may benefit from Toftness CMT and that the data justified a clinical trial be undertaken. During the study, no side effects or deterioration of clinical presentations were noted among the pediatric participants [46].

A second high quality RCT investigated the use of OMT for 57 children with acute otitis media. In this study, Mills et al. grouped 25 participants into the treatment group that received OMT in addition to routine pediatric care and 32 subjects in the control group who received only routine pediatric care. The average age was 26 months in the treatment group and 20 months in the control group. Decreased symptoms and improved tympanogram scores were only reported in the OMT group. The researchers stated there were no adverse events reported during the study [47].

*Overall Summary:*

***Inconclusive (favorable)*** *evidence for OMT in treating acute otitis media.*

***Inconclusive (unclear)*** *evidence for CMT (Toftness technique) in treating acute otitis media.*

4. Special needs

Table 9 summarizes the ten studies investigating the use of manual therapy for pediatric conditions categorized as special needs that met our inclusion criteria. One study investigated OMT on children with Attention Deficit Hyperactive Disorder (ADHD) [48], two studies investigated the use of manual therapy for autistic children [49, 50], (one used VOMT and the other used CMT). Three studies investigated the use of OMT on children with cerebral palsy [51–53] and four of the studies investigated the use of OMT on premature infants [54–57].

4.1. Attention deficit hyperactive disorder (ADHD)

**Table 9:** Data extraction for the special needs studies

Condition	Author/year	Study objective	Study design sample size intervention	Patient description/condition	Primary/main outcome(s)	Main results/conclusions	Adverse events
ADHD	Accorsi A, et al. 2014 [48]	To evaluate efficacy of osteopathic manipulative treatment of children with ADHD.	RCT *n* = 28 OMT	Children ages 5–15, with primary diagnosis of ADHD	Biancardi-Stroppa Modified Bell Cancellation Test, accuracy and rapidity scores	Osteopathic manipulative treatment was positively associated with changes in the Biancardi-Stroppa Test accuracy and rapidity scores.	There was no mention of adverse events made in this study.
Autism	Bramati-Castellarin I, et al. 2016 [49]	Investigate the influence of visceral osteopathic technique on the behaviour and GI symptoms of children with autism.	Interrupted Time Series (without control group) *n* = 49 VOMT	Autistic children ages 3 1/2–8, with GI symptoms, impaired social interactions and communication	Parental completion of the Modified Autism Research Institute outcomes survey form (9 S.O.S. questionnaires) and secretin assessment used to assess GI signs and symptoms	Significant improvements reported in “social behavior and communication” and “digestive signs” subscale of the questionnaire and in vomiting and poor appetite comparing before and after VOMT.	“There was no mention of adverse events made in this study.”
	Khorshid K, et al. 2006 [50]	Identify the differences in efficacy between upper cervical and full spine adjustment in autistic children	RCT *n* = 14 CMT	Children diagnosed with autism	ATEC average scores and parental observations	Clinical improvements observed through parental observations and through a decrease in the ATEC scores in both groups. Upper cervical group had improved ATEC average scores of 32%. Full spine group had improved ATEC scores of 19%.	Clinical deterioration was shown in one of the children of the full spine group, but only marginal in one child of the upper cervical group.
Cerebral Palsy	Wyatt K, et al. 2011 [51]	Evaluate the general health and wellbeing effect of cranial osteopathy on cerebral palsy children.	RCT *n* = 142 Cranial Osteopathy	Children with CP, ages 5–12	Gross Motor Function Measure - (GMFMM-66) Quality of life Child Health Questionnaire-(CHQ) PF50	No statistical change in GMFM-66 or CHQ. PF50 Parents (unblinded) reported better global health.	No serious adverse events were reported.
	Duncan B, et al. 2004 [52]	Evaluate effectiveness of osteopathic manipulation or acupuncture as a supplemental therapies for children with spastic cerebral palsy.	RCT *n* = 50 OMT	Children with spastic CP, ages 20 months - 12 years	Parent reporting of changes observed (open-ended questions)	96% reported improvements. Most frequent seen in use of arms and legs (61 and 68%) and more restful sleep (39 and 68%) in osteopathic and acupuncture respectively. Additional improvements also noted in mood and bowel functions.	There was no mention of adverse events made in this study.
	Duncan B, et al. 2008 [53]	Evaluate effectiveness of osteopathic manipulation (cranial field, myofascial release or both) vs. acupuncture in spastic cerebral palsy patients.	RCT *n* = 55 OMT/Acupuncture	Children with CP, ages 20 months - 12 years	11 outcomes used: Primary-GMFCS, GMFM total percent, PEDI mobility, PEDI self-care, WeeFIM mobility, WeeFIM self-care; Secondary- DO rating of spasticity, MAS biceps, MAS hamstring, parent/guardian rating of arched back, parent/guardian rating of startle reflex	Osteopathic manipulation was associated with improvements in 2 of 11 outcomes; GMFM total percent and WeeFIM Mobility. Acupuncture was not associated with improvements in any of the outcomes variables.	There was no mention of adverse events made in this study.
Prematurity	Raith W, et al. 2016 [54]	Investigate neurological short term effects of craniosacral therapy on general movements in preterm infants.	RCT *n* = 30 OMT/CST	Preterm infants ages 25–33 weeks, free from medical complications in NICU	Primary outcome: General movement assessment tool. Secondary outcomes: General movement optimality score	No difference in the general movement could be observed between the groups. No change in general movement optimality score was noted.	There was no mention of adverse events made in this study.
	Cerretelli F, et al. 2015 [55]	Investigate whether osteopathic manipulation reduces the length of hospital stay, costs, and weight gain for preterms.	RCT *n* = 695 OMT/CST	Preterm infants ages 29–37 weeks, without congenital complications in NICU	1. Reducing length of hospital stay 2. Weight gain and hospital savings	Osteopathic treatment reduced days hospital (3.9 days) and reduced costs by 1250.65€ per newborn per length of stay. No change in weight gain was noted.	There were no complications associated to the intervention.
	Pizzolorusso G, et al. 2014 [56]	Investigate whether osteopathic manipulation reduces length of hospital stay, what effect the timing of introduction of osteopathic treatment may have on the outcome and hospital costs in preterm infants.	RCT *n* = 110 OMT/CST	Preterm infants ages 32–37 weeks, free from medical complications in NICU	1. Reducing length of hospital stay and impact on length of stay of timing of introduction of osteopathic manipulation 2. Reducing hospital cost	Sooner osteopathic manipulation introduced, shorter length of stay. There is a positive association of osteopathic manipulation with overall reduction in cost of care.	There were no complications associated to the intervention.
	Cerretelli F, et al. 2013 [57]	Determine effectiveness of osteopathic manipulative therapy in reducing the length of hospital stay, hospital costs and weight gain in preterm infants.	RCT *n* = 110 OMT/CST	Preterm infants ages >28 and <38 weeks, free from medical complications in NICU	1. Decreased length of hospital stay 2. Improved weight gain and reduced NICU costs	Osteopathic manipulation reduced length of stay and hospital costs but not effect weight gain.	No serious adverse events were reported.

One study was found that investigated the use of OMT on patients with ADHD [48].

Accorsi et al. conducted a high quality RCT evaluating the efficacy of OMT in the treatment of 28 children ages 5 to 15 years old with ADHD. One half of the participants (*n = 14*) were placed in a treatment group, which received OMT plus conventional treatment, and one half of participants *(n = 14)* were placed in the control group, receiving conventional therapy alone. The outcome measures were better accuracy and rapidity scores on the Biancardi-Stroppa Modified Cancellation test, a test that is used to measure visual-spatial attention. Accorsi et al. reported the children in the intervention group demonstrated statistically significant improvement in selective and sustained attentive performances, as measured using the Biancardi-Stroppa Modified Cancellation Test. These findings prompted the researchers to recommend a larger study be undertaken. There is no mention of adverse events in this study [48].

*Overall Summary:*

***Inconclusive (favorable)*** *evidence for OMT in treating ADHD in children.*

4.2. Autism

Two studies were found that investigated the use of manual therapy on patients with autism. One looked at the use of visceral osteopathic manual therapy (VOMT) the other CMT [49, 50].

A medium quality interrupted time-series without comparison was conducted by Bramati-Casterllarian et al. They investigated the influence of VOMT on behavior and GI symptoms on children with autism. Their study included 49 autistic children ages 3 1/2 to 8 years of age with GI symptoms and impaired social interactions and communication. The primary outcome measure they utilized was parental completion of the Modified Autism Research Institute survey and secretin assessment to assess the GI signs and symptoms. Overall significant symptomatic improvement for social behaviors and communication, as well as improvement in digestive issues such as vomiting and poor appetite, were reported. They concluded VOMT could have a significant improvement in quality of life and well-being for children suffering from both autism and GI signs and symptoms. There was no mention of adverse events made in this study [49].

A low quality RCT without a control group intended to identify the differences in efficacy between Upper Cervical CMT and Full Spine (Diversified) CMT in 14 autistic children. The clinical effects of the autistic children were evaluated using the Autism Treatment Evaluation Checklist, a questionnaire that assessed the children’s development and progress that is answered by the parents. Although autistic children in both groups demonstrated improvements in their autistic behaviours, the ATEC score for the upper cervical group was 32% versus 19% for the full spine group. The authors concluded autistic children receiving Upper Cervical CMT experienced better improvement in their autistic behaviors compared to autistic children receiving Diversified CMT. There is no mention of adverse events in this study [50].

*Overall Summary:*

***Inconclusive (unclear)*** *evidence for VOMT in treating autism.*

***Inconclusive (favorable)*** *evidence for CMT in treating autism.*

4.3. Cerebral palsy

Three RCT’s were found that met our criteria investigated the use of OMT on children with cerebral palsy [51–53].

A high quality pragmatic RCT evaluated the effect of OMT using cranial therapy on the general health and well-being of 142 children ages 5–12 with cerebral palsy. In this study, Wyatt et al. placed 71 children in treatment group, who received 6 OMT sessions over 6 months and 71 children in a control group, which they referred to as “waiting list”. Primary outcome measures included: Gross Motor Function Measure 66 (GMFMM-66) and Quality of Life Child Health Questionnaire (CHQ) PF50. Secondary outcomes measures used in this study included: Parental Assessment of Global Health and Sleep at 6 months, Pain and Sleep Questionnaire at 10 weeks and 6 months, CHQ PF50 at 10 weeks and the Quality of Life Short Form-36. This trial showed no statistically significant evidence that OMT led to sustained improvement in motor function, pain, sleep, quality of life of the subjects or in the quality of life of their caretakers. No serious adverse events were reported and none of the children withdrew from the study due to side effects of the treatment [51].

Duncan et al. conducted a high quality assessor blinded wait-list controlled pilot RCT that investigated the effectiveness of OMT (cranial therapy), myofascial release or both versus acupuncture on 55 cases of children ages 20 months to 12 years with moderate to severe spastic cerebral palsy. Participants were grouped into one of three groups: OMT (which included osteopathy, myofascial release or both) (*n* = 15), acupuncture (*n* = 19) and wait-list control (non-therapeutic attention) (*n* = 22). The six primary outcome measures were: Gross Motor Functional Classification, Gross Motor Measurement Total percentage, Pediatric Evaluation of Disability Inventory mobility and self-care, and Functional Independent Measure for Children mobility and self-care. Duncan et al. reported that OMT resulted in an improvement in the child’s gross motor function as indicated by the outcome measures in children with moderate to severe spastic cerebral palsy. There was no mention of adverse events in this study [53].

A low quality RCT evaluated the effectiveness of OMT, acupuncture or both for 50 children aged 11 months to 2 years with spastic cerebral palsy. Participants were grouped into four groups: OMT (*n* = 23), acupuncture (*n* = 19), both OMT and acupuncture (*n* = 8) and wait-list control (*n* = 19). Multiple outcome variables were used to determine if these interventions would decrease muscle tone, improve function and quality of life. Evaluation in this study included parental interviews to assess perceptions and changes observed. Only 2 of 17 parents reported positive gains while their child was in a wait-list control period, but all 17 parents reported gains while in the treatment phase of the study. Improvement was claimed by 96% (48 of 50) of the parents while their child was receiving treatments, but the gains varied. The most frequent gains were seen in improvement in the use of arms or legs (61 and 68%) and more restful sleep (39 and 68%) in the OMT and the acupuncture groups, respectively. Improvement in mood and improved bowel function were also very common benefits noted by the parents in both groups. There is no mention of adverse events in this study [52].

*Overall Summary:*

***Inconclusive (unclear)*** *evidence for OMT in treating children with cerebral palsy.*

4.4. Prematurity

Four high quality RCTs were found, that investigated the use of OMT on various clinical outcomes of children born preterm [54–57].

A high quality RCT was conducted by Raith et al. on 30 preterm infants between 25 and 33 weeks in the neonatal intensive care unit, free from medical complications, with OMT/CST. The aim was to investigate neurological short term effects of craniosacral therapy on general movement in preterm infants. The primary outcome utilized was improvement in general movement assessment tool. Secondary outcomes included improvement in general movement optimality score. They found no differences between the control or study group for all outcome measures and at all time points. There was no mention of adverse events made in this study [54].

Cerretelli et al. conducted a high quality RCT in 2015 that investigated the effectiveness of OMT/CST on length of hospital stay, hospitalization costs, and weight gain in 695 preterm infants’ ages 29–37 weeks. (Study group, *n* = 352; control group, *n* = 343) The primary objective was in determining the effect of OMT/CST in reducing the length of the hospital stay. Secondary objectives evaluated the effect on weight gain and NICU cost savings. They found a reduction in days in hospital (3.9 days) and associated cost savings, but no significant change in weight gain after OMT/CST compared to the control group. Similar to the Pizzolorusso et al. 2014 study, the description of the intervention listed as “manipulation” met the characteristics of cranial/craniosacral therapy. No complications were associated with the intervention [55].

Pizzolorusso et al. investigated whether OMT (cranial sacral) reduced the length of the hospital stay in 110 preterm infants ages 32–37 weeks in a high quality RCT. Fifty-five infants were placed in the study group who received routine pediatric care and OMT/CST and compared to 55 infants in the control group who received routine pediatric care only. The primary objective of the study was to determine the effect of OMT/CST on reducing the length of stay and what effect the timing of introduction OMT/CST may have on the outcome. The secondary objective was to estimate the potential savings in terms of hospital costs. Pizzolorusso et al. reported that length of stay and neonatal intensive care unit costs were improved after introduction of OMT. It was also concluded that the earlier the OMT/CST had the shorter the hospital stay. No adverse events were recorded in this study [56].

Lastly, Cerretelli et al. conducted another high quality RCT that sought to determine the clinical effects of OMT in 110 preterm infants ages range 29–37 weeks. The treatment group had 55 assigned to receive OMT/CST plus routine pediatric care. They were compared to 55 infants in the control group who received only routine pediatric care. The primary outcome measure was to determine the effectiveness of OMT/CST in reducing the length of the hospital stay. Secondary objectives included determining the effect of OMT/CST on weight gain and in reducing NICU costs. The results of this study show that OMT reduced the length of stay (− 5.9 days) and NICU costs, but did not impact weight gain. They suggested that further studies based on multi-center design are required to confirm these results. No adverse or side effects were shown in either group [57].

*Overall Summary:*

***Moderate (favorable)*** *evidence for OMT/CST in reducing length of stay and hospital costs for preterm infants.*

***Inconclusive (unclear)*** *evidence for OMT/CST in improving general movement in preterm infants.*

5. Structural conditions

Table 10 provides a summary of ten studies that were categorized as “structural” conditions. Two studies assessed changes to cranial asymmetry [58, 59], one evaluated postural asymmetry [60], five studies investigated scoliosis [61–65], one study evaluated torticollis [66], and one study evaluated upper cervical dysfunction [67].

5.1. Cranial asymmetry (non-synostotic plagiocephaly)

**Table 10:** Data extraction for structural studies

Condition	Author/year	Study objective	Study design sample size intervention	Patient description/ condition	Primary/main outcome(s)	Main results/conclusions	Adverse events
Cranial Asymmetry	Cabrera-Martos I, et al. 2016 [58]	Evaluate the effects of manual therapy as an adjuvant option on treatment duration and motor development in infants with severe nonsynostotic plagiocephaly.	RCT *n* = 46 MT/CST	Infants ages 4–8 months, with severe nonsynostotic plagiocephaly	Treatment duration and motor development assessed with Alberta Infant Motor Scale	Treatment duration was significantly reduced in manual therapy group (109.84 +/− 14.45) compared to the control group (148.65 +/− 11.53) days. Asymmetry after the treatment was minimal Type 0 or Type 1. Motor behaviour was normal in all the infants after treatment.	Study reported no adverse effects were seen during the treatment period.
	Lessard S, et al. 2011 [59]	Does osteopathic manipulation alter cranial asymmetry in infants.	Before-After *n* = 12 OMT	Infants ages < 6.5 months, diagnosed with nonsynostotic plagiocephaly	Anthropometric changes	Osteopathic treatment led to improvements in cranial asymmetry.	There is no mention of adverse events made in this study.
Postural Asymmetry	Philippi H, et al. 2006 [60]	To assess the therapeutic efficacy of osteopathic manipulation in infants with postural asymmetry.	RCT *n* = 32 OMT/CST	Infant ages 6–12 weeks, with postural asymmetry	Video-based measurements	Significant improvement in postural asymmetry (mean 5.9 points) observed with osteopathic manipulation.	“At least two of the seven vegetative symptoms aggravated for 2 days after the interventions in six patients of the control group and in four patients of the treatment group. Otherwise no adverse effects were seen.”
Scoliosis	Byun S & Han D, 2016 [61]	Examine whether chiropractic techniques would reduce the curvature of idiopathic scoliosis.	Before-After *n* = 5 CMT	Children ages 10–13, with Cobb angles > 10 degrees	Reduction in Cobb angle	No significant difference in Cobb angle was noted after the 4th week of chiropractic manipulation.	There is no mention of adverse events made in this study.
	Hasler C, et al. 2010 [65]	Test to see if osteopathy alters trunk morphology, to unload the concave side of the scoliosis to halt curve progression.	RCT *n* = 20 OMT	Post-pubertal females ages 12–18, with Cobb angles 20–40	Trunk morphology, spine flexibility and scoliometer measurements	Repeat measurements revealed no therapeutic effect on rib hump, lumbar prominence, plumb line, sagittal profile and global flexibility.	“No intervention-related side effects or complications were noted”
	Rowe D, et al. 2006 [62]	To conduct a pilot (feasibility) study and explore issues of patient safety, patient recruitment and compliance, treatment standardization, sham treatment refinement, interprofessional cooperation, quality assurance, and outcome measure selection.	RCT *n* = 6 CMT	Children ages 10–16, with Cobb angles 20–40 degrees	Reduction in Cobb angle	Feasible to recruit AIS patients for a randomized clinical trial to compare chiropractic care and standard medical treatment.	CMT delivered on 52 visits resulted in two benign reactions one with moderate pain lasting 24 h; the other produced mild pain lasting 6 h.
	Morningstar M, et al. 2004 [63]	Evaluate of scoliosis treatment using a combination of manipulative and rehabilitative therapy.	Before-After *n* = 19 (6 pediatrics) CMT	Scoliotic patients aged 15–65 (6 patients 18 and under- identified in Table 3 of study)	Reduction in Cobb angle	Reduction in Cobb angles in all patients.	There is no mention of adverse events made in this study.
	Lantz C & Chen J, 2001 [64]	Effect of chiropractic manipulation on small scoliotic curves in younger subjects.	Before-After *n* = 42 CMT	Children aged 6–17, with Cobb angles 6–25	Reduction in Cobb angle	No overall reduction in Cobb angle after 6.5–28.5 months of care.	There is no mention of adverse events made in this study.
Torticollis	Haugen E, 2011 [66]	Evaluate measurement methods and examine short-time effect of manual therapy in addition to physiotherapy in infants with torticollis.	RCT *n* = 32 MT	Infant aged 3–6 months, diagnosed with torticollis	Primary outcome: Videoclip recordings, Secondary outcomes: 12 parameters of body function, activity, participation	No significant difference in primary outcome. Found non-significant tendency to greater improvement in lateral flexion and head righting in intervention group.	There is no mention of adverse events made in this study.
Upper Cervical Dysfunction	Saedt E, et al. 2018 [67]	To gain insight into the patient characteristics and reasons for seeking care in infants with indications of upper cervical dysfunction referred for manual therapy.	Before-After *n* = 295 MT	Infants aged < 27 weeks, with positional preference, restlessness, abnormal head position, excessive crying	Improved flexion-rotation test and lateral flexion tests Parental perception of treatment effects Pre- and post treatment self-reported questionnaires	Flexion- rotation test decreased from 78.8 to 6.8%. Lateral flexion test decreased from 91.5% tp 6.2%. All parents perceived positive treatment effects.	No serious adverse events were reported during this study.

Two studies investigated the use of manual therapy on cranial asymmetry.

One high quality RCT evaluated the use of MT/CST [58], the other a medium quality before-after observational study looked at OMT [59].

Cabrera-Martos conducted a high quality RCT that evaluated the effects of CST in infants with severe nonsynostotic plagiocephaly. Forty-six children meeting eligibility were randomized into control and study groups. Twenty-three children allocated to the control group received standard treatment which included positional changes and the use of an orthotic helmet. The study group included 23 infants who received CST in addition to standard treatment to evaluate treatment duration and motor development. The primary outcome utilized was the Alberta Infant Motor Scale at baseline and at discharge of the patients. The results of the study showed that CST added to usual treatment for severe nonsynostotic plagiocephaly resulted in significant improvement in asymmetry, less treatment duration, and improved motor behavior. There were no adverse events seen during the treatment period [58].

One medium quality pilot before-after study reported favorable results utilizing OMT (the most frequently used techniques used in the study were described as “cranial” work) on 12 infants with cranial asymmetry. Twelve infants with cranial asymmetries received four OMT treatments over 2 weeks. Anthropometric, plagiocephalometric, and qualitative measures were administered pre-intervention, during the third treatment and 2 weeks after the fourth treatment. The study group showed a significant decrease in cranial vault asymmetry, skull base asymmetry, and trans-cranial vault asymmetry. The researchers concluded that OMT contributes to improvements in cranial asymmetries in infants younger than 6.5 months presenting with nonsynostotic occipital plagiocephaly characteristics. There was no mention of adverse events in this study [59].

*Overall Summary:*

***Inconclusive (favorable)*** *evidence for both OMT and MT/CST in treating cranial asymmetry in children.*

5.2. Postural asymmetry

One high quality RCT reported improved infant postural asymmetry utilizing OMT/CST on 32 infants, (18 males, 14 females) with gestational age of at least 36 weeks. Infants were assigned to intervention (*n* = 16) or sham (*n* = 16) groups. Outcomes were measured using a standardized video-based asymmetry scale from baseline to final visit. In the control group, the mean improvement was 1.2 points. In the treatment group, the mean improvement was 5.9 points. The researchers concluded that OMT/CST in the first months of life is beneficial for infants with idiopathic asymmetry. At least two of the seven vegetative symptoms aggravated for 2 days after the intervention in six patients of the control group and in four patients of the treatment group. No other adverse events were described [60].

*Overall Summary:*

***Inconclusive (favorable)*** *evidence for OMT/CST in treating postural asymmetry in children.*

5.3. Scoliosis

Five studies looked at the use of manual therapy in the treatment of scoliosis. Four looked at the use of CMT [61–64]. Of these, one was a high quality RCT, three before-after, two medium and one of low quality. The fifth study was a high quality RCT that looked at the use of OMT [65].

A medium quality before-after observational study by Byun and Han examined whether chiropractic techniques would reduce the curve of adolescent idiopathic scoliosis in 5 healthy children with an average age of 11.8 years with Cobb angles greater than 10 degrees (average 11.2 degrees). The primary outcome was the change in the Cobb angle that was measured after 4 and at 8 weeks of treatment. The results of this study were that the Cobb angle was noticeably decreased after 4 weeks, but no further reduction in Cobb angle was noted after 8 weeks, except in one male. They concluded that chiropractic techniques effectively reduced the Cobb angle in adolescent idiopathic scoliosis after 4 weeks. There was no mention of adverse events made in this study [61].

Rowe et al. conducted a high quality pilot RCT that investigated the clinical effects of CMT on children with scoliosis. This was a feasibility study whose purpose was to explore issues of safety, patient recruitment, patient compliance, treatment standardization, sham treatment refinement, inter-professional cooperation, quality assurance, and outcome measure selection. The primary outcome measured was the Cobb angle. Secondary outcome was the Scoliosis Quality of Life Index (SQLI). The researchers reported improved Cobb angles in 5 of the 6 patients that received CMT and an improved SQLI in 1 of the 6. Due to the small sample size, no conclusions could be made regarding effectiveness. Regarding adverse events, CMT delivered on 52 visits resulted in two benign reactions; 1 with moderate pain lasting 24 h, the other produced mild pain lasting 6 h [62].

Morningstar et al. conducted a low quality before-after case series that reviewed the clinical files of 22 patients, 6 of whom were 18 years or younger, who received a combination of CMT and rehabilitative therapy. The authors found reductions in Cobb angle (average 17 degree reduction) in all the patients, including the patients under the age of 18 years. No mention of any adverse events was noted in this study [63].

Lantz et al. conducted a medium quality before-after case series of 42 children, 16 males, 26 females, with scoliotic curves ranging from 4 to 22 degrees, ages 6–17 years, to determine the clinical effects of full spine CMT, use of heel lifts, and lifestyle counseling on the progression of the curves. Participants were treated for between 6.5 to 28.5 months. The main outcome was a reduction in scoliotic curvature. The authors reported no overall improvement in scoliotic curves using CMT. No mention of adverse events was made in this study [64].

Hasler et al. conducted a high quality prospective RCT that sought to determine if OMT altered trunk morphology to unload the concave side of the scoliosis in order to halt curve progression. The study included 20 pre-pubertal women with curves that ranged from 20 to 40 degrees. The primary outcomes was trunk morphology and spine flexibility. The authors concluded that there was no evidence to support the use of OMT in the treatment of mild idiopathic scoliosis. No intervention-related side effects or complications were noted [65].

*Overall Summary:*

***Inconclusive (unclear)*** *evidence for use of CMT in childhood scoliosis.*

***Inconclusive (unfavorable)*** *evidence for use of OMT in childhood scoliosis.*

5.4. Torticollis

One medium quality pilot RCT investigated whether MT improved torticollis in 32 patients between the ages of 3–6 months. There were 15 infants in the study group who received MT plus physiotherapy (PT) and 16 infants in the control group who received child physiotherapy alone. The study did not describe the type of MT provided. The primary outcome measured was evaluating the torticollis symptoms via videotape footage of the child using a 4-point scale in which the child was rated as “much better”, “better”, “no significant change” or “worse”. Secondary outcomes included 12 measurement parameters that involved body function, activity, and participation corresponding to the International Classification of Function The study reported no significant improvement in the MT and PT group in the primary outcome, but improvement in two of the secondary outcome measures of improved passive and active lateral flexion of the neck. No mention of adverse events were noted in this study [66].

*Overall Summary:*

***Inconclusive (unfavorable)*** *evidence for MT for torticollis.*

5.5. Upper cervical dysfunction

A high quality before-after observational study by Saedt et al. sought to gain insight into the patient characteristics and reasons for seeking care in infants with upper cervical dysfunction (UCD). A group of 295 infants (mean age of 11.2 weeks) with positional preference, restlessness, abnormal head position and excessive crying were treated with mobilization. The primary outcomes were assessed with pre- and post-treatment self-reported questionnaires used to assess diagnostics, treatment procedures, outcomes, and harms from parents and manual therapists. The questionnaires consisted of two sections: one collected at baseline; the other posttreatment by both the parents and the manual therapists. The researchers concluded that the majority of infants with upper cervical dysfunction showed positional preference of the head and reduced the active and passive mobility of the upper cervical spine. After gentle upper cervical mobilization techniques, active and passive cervical mobility increased. They also reported that the parents reported a reduction in symptoms. No serious adverse events were reported during this study [67].

*Overall Summary:*

***Inconclusive (unclear)*** *evidence for the use of MT in infants with upper cervical dysfunction.*

## Discussion

This review identified 50 RCTs and observational original studies that evaluate manual therapy for pediatric conditions, which updates several previously published systematic reviews. Of particular importance, our review included studies investigating the effects of manual therapy on musculoskeletal conditions, including pediatric low back pain and headache. Other conditions not previously reported in some previous systematic reviews include: constipation, suboptimal infant breastfeeding, clubfoot, cuboid syndrome, headache, pulled (nurse’s) elbow, asthma, obstructive apnea, autism, cranial asymmetry, postural asymmetry, scoliosis, torticollis, and upper cervical dysfunction.

Of the 50 studies, 32 were RCTs (18 high-quality, 10 medium-quality, and 4 low-quality). The remaining 18 studies were observational (1 high-quality, 13 medium-quality, and 4 low-quality). Observational studies were further broken down by study design (13-before-after, 4 interrupted time-series without comparison group, and 1 interrupted times-series with comparison group). Thirty-six studies reported ‘favorable’ results, five showed ‘no improvement’, and nine showed ‘no difference’ between study groups. In five of the nine ‘no difference’ studies, ‘favorable’ results were noted in both groups, of which two of these studies had MT in both groups.

Pediatric conditions assessed as ‘moderate-favorable’ were:

- Low Back Pain (using CMT);
- Pulled (or Nurse’s) Elbow (using MT); and
- Preterm Infants (using OMT/CST to reduce days and costs in hospital).

Pediatric conditions assessed to be ‘inconclusive- favorable’ were:

- ADHD (using OMT);
- Autism (using CMT);
- Asthma (using OMT);
- Clubfoot (using MT);
- Cranial Asymmetry (using MT/CST);
- Dysfunctional Voiding (using OMT);
- Infantile Colic (using OMT/CST);
- Obstructive Apnea (using OMT);
- Otitis Media (using OMT);
- Postural Asymmetry (using OMT/CST);
- Suboptimal Infant Breastfeeding (using CMT/CST); and
- Temporomandibular Joint Dysfunction (using OMT).

Pediatric conditions assessed to be ‘inconclusive-unclear’ were:

- Asthma (using CMT);
- Autism (using VOMT);
- Cerebral Palsy (using OMT);
- Constipation (using OMT);
- Cranial Asymmetry (using OMT);
- Cuboid Syndrome (using MT);
- Headache (using CMT, OMT, and MT);
- Infantile Colic (using CMT);
- Low Back Pain (using MT);
- Otitis Media (using CMT);
- Nocturnal Enuresis (using CMT);
- Preterm Infants (using OMT/CST for general movement);
- Scoliosis (using CMT); and
- Upper Cervical Dysfunction (using MT).

Pediatric conditions assessed to be ‘inconclusive-unfavorable’ were:

- Scoliosis (using OMT) and
- Torticollis (using MT).

Our findings had a few notable updates from prior systematic reviews, especially the UK Update, of which “inconclusive-unclear” or “inconclusive-favorable” was the outcome for all conditions [10]. The UK Update was unable to review any musculoskeletal conditions because no studies were available at that time [10]. Evans et al. published the first high-quality RCT on adolescent low back pain, which allowed for this review to report a “moderate-positive” evidence for low back pain using CMT [33]. Another musculoskeletal condition that has an ongoing study is headaches (ClinicalTrials.gov Identifier: NCT02684916); we anticipate the results of this study will allow for better practitioner guidance because of the high rigor described in the protocol. Pulled (Nurse’s) elbow using MT was also not in the UK Update, and was found to have a “moderate-positive” result in this study [37, 38].

Additional evidence ratings changed in a positive direction in our study from the UK Update for preterm infants (reducing length of stay and hospital costs) using OMT/CST. Three new high-quality RCT’s not previously identified by the UK Update were identified showing favorable results, which accounted for this modification [55–57]. We were able to change the evidence ratings from “inconclusive-unclear” to “inconclusive-favorable” for two additional conditions: otitis media, based on data gathered from two medium quality RCT’s [43, 45], reporting favorable results and for ADHD, based on the results of a high quality RCT showing favorable results [48].

We amended the evidence from “inconclusive-favorable” to “inconclusive-unclear” for infantile colic and pediatric enuresis using CMT. Regarding the change for infantile colic, our study included four studies, the most recent of which is a high-quality with improved outcomes [19]. However, the remaining studies showed either “no improvement” or “no difference” [20–22]. Our evidence rating is similar to the recent 2018 systematic review and meta-analysis of infantile colic and manual therapy conducted by Carnes et al. [68]. Carnes et al. concluded that while small benefits were found for the overall outcome, the benefit of manual therapy for infantile colic is still unclear [68]. For pediatric enuresis, our search identified only one observational study showing favorable results; however, this level of evidence was not enough to substantiate a “favorable” rating [25]. The UK Update conclusion was based off the Huang et al. systematic review, which included clinical trials that did not meet our eligibility criteria for manual therapy and year of publication [69].

Similar to the previous systematic review on this topic, and despite using only recent literature, this review continued to find serious methodological limitations within the included studies. Our most common methodological concern was the lack of standardization of the intervention, which varied across the professions and even between studies within the same profession. Many studies failed to adequately describe the methods used by the practitioner; most of the studies also failed to describe the number of treatments the patients received and over what duration of time. In addition, the provider’s experience, training, and type of intervention used in the same study varied considerably. Another notable methodological concern was small sample size, which was not accounted for in the quality assessment. Finally, many studies failed to report on the incidence of adverse events.

Adverse events were addressed in only 20 of the 50 included studies reviewed. No lasting or significant adverse events were reported for children receiving any form of MT. Two previous systematic reviews have been published regarding the incidence of adverse events associated with pediatric spinal manipulation [7]. These reviews report that adverse events are rare, but that the true incidence is unknown as they have not been evaluated prospectively. The current “Best Practices for Chiropractic Care of Children: A Consensus Update” report states that the best way to minimize adverse events is by conducting a thorough history and examination, as the majority of adverse event cases in the literature are often due to underlying pre-existing pathology that was not diagnosed [9]. Our review is in agreement with previous studies in recommending that prospective-population-based studies should be conducted to identify the true incidence of serious adverse events due to MT in the pediatric population. Such a clinical trial is currently ongoing (ClinicalTrials.gov Identifier: NCT02268331).

Additionally the “Best Practice” report states that manual procedures should be modified when treating children to take into account the differences in patient size, structural development and flexibility of the joints [9]. Modifications should include using gentler, lighter biomechanical forces proportioned to the size and structural development of the child. Both Triano et al. and Todd et al. [8, 70]. have posited that healthcare providers using SMT are able to modulate the amount of forces used. We agree this ability to modulate for pediatric, geriatric, and other special populations ought to be included in undergraduate training programs or during continuing education workshops for field practitioners.

### Limitations

Aside from using rigorous methodology in this systematic review and conducting a comprehensive search, it is possible that our search failed to identify every relevant study, especially considering the restriction of the search to English-language studies. Our knowledge of unpublished trials have influenced our conclusions; unpublished trials may be more likely to produce negative or equivocal results. Although the independent reviewers performed this review, and in spite of utilizing systematic strategies for assessing the quality of the included studies, there is still room for subjective interpretation. While we deliberately chose widely accepted recommendations for assessing quality and determining bias, our adaptation of some recommendations to better fit our study design may have impacted our conclusions. Also, each reviewer has varying degrees of familiarity with the assessment tools *a priori*, which could influence the inter-reviewer reliability of the primary quality and bias assessments. Finally, all six reviewers are chiropractors; this expertise, as well, may be considered a source of bias.

## Conclusions

Favorable, albeit inconclusive, results were reported in 36 of the 50 studies we assessed that used different types of manual therapies for pediatric conditions. Compared to previous reviews of the literature, we found a number of clinical trials investigating the effects of manual therapies on pediatric musculoskeletal conditions. Twenty-four studies included information on adverse events that were all transient and mild to moderate in nature. Clearly more research investigating the clinical effectiveness of manual therapies for pediatric conditions, along with the incidence of adverse events, is required in order to allow practitioners and parents to make better informed choices with respect to care planning for children with pediatric conditions.

## Abbreviations

ADHD: Attention deficit hyperactivity disorder
AHRQ: Agency for Healthcare Research and Quality
CAM: Complementary and alternative medicine
CMT: Chiropractic manipulative therapy
CST: Cranial-sacral therapy
EENT: Eyes, ears, nose, and throat
HHS: Department of Health and Human Services
HVLA: High velocity, low amplitude
LBP: Low back pain
MT: Manual therapy
NHIS: National Health Interview Statistics
OMT: Osteopathic manual therapy
RCT: Randomized controlled trial
SMT: Spinal manipulation therapy
TMD: Temporomandibular dysfunction
UK: United Kingdom
US: United States
VOMT: Visceral osteopathic manipulation
